# A Reproducible Post-Valve-Replacement EHR Cohort for Comparative AI Studies

**DOI:** 10.3390/diagnostics16030447

**Published:** 2026-02-01

**Authors:** Malte Blattmann, Mika Katalinic, Adrian Lindenmeyer, Stefan Franke, Thomas Neumuth, Daniel Schneider

**Affiliations:** Innovation Center Computer Assisted Surgery (ICCAS), Leipzig University, Semmelweisstrasse 14, 04103 Leipzig, Germany

**Keywords:** data preprocessing, machine learning, electronic health records (EHR), personalized medicine, perioperative care

## Abstract

**Background/Objectives**: Valve replacement (VR) patients are at high risk of postoperative complications, but reproducible Electronic Health Record (EHR) benchmarks for evaluating sequential AI models in this setting are lacking. We develop a reproducible pipeline that extracts two EHR datasets from MIMIC-IV (a general-purpose and a predictive benchmark dataset) capturing perioperative histories, high-resolution time-series, and clinically motivated outcome labels. **Methods**: The cohort comprises 3890 VR patients with clinician-guided feature selection across diagnoses, procedures, laboratory measurements, medications, and physiological monitoring. As an exemplary use case, we define ICU readmission at first ICU discharge as a surrogate for postoperative risk and derive a predictive benchmark under strict label-leakage control. We then compare a Transformer model trained on tokenized longitudinal EHR sequences with Transformer and XGBoost baselines trained on aggregated feature statistics, and assess performance differences using paired statistical tests across validation splits. **Results**: ICU readmission stratified in-hospital and 100-day outcomes, including mortality, complications, and rehospitalization, confirming the clinical relevance of the prediction target. The sequential Transformer achieved 0.87 AUROC and 0.69 AUPRC. Corrected resampled *t*-tests confirm improved performance over the non-sequential Transformer, while the comparison with XGBoost indicates a favorable trend without conclusive evidence. **Conclusions**: Our findings suggest that leveraging longitudinal EHR sequences yields higher predictive performance than static feature summaries for postoperative risk prediction. The publicly released preprocessing pipeline and cohort-construction code enable researchers with MIMIC-IV access to reproduce the datasets and provide a robust benchmark for developing and comparing time-series models in post-valve replacement care.

## 1. Introduction

Valve replacement (VR) surgery is a major intervention for patients with heart valve disease and carries a substantial risk of severe complications, including thromboembolic events, infections, and prosthesis-related mechanical failure [1,2,3]. Such events can considerably impair recovery and long-term outcomes. Electronic health records (EHRs) offer an opportunity to improve perioperative management by enabling earlier risk stratification and targeted interventions (e.g., enhanced monitoring or personalized postoperative care). Consistent with this, structured perioperative care protocols in cardiac surgery have been associated with shorter hospital stays, lower mortality, and fewer complications [4,5].

Despite this potential, the practical use of EHRs for developing and evaluating predictive models remains challenging. EHR data are heterogeneous and irregular, which limits the applicability of conventional statistical approaches [6], and their scale makes manual review infeasible [7]. Machine learning (ML), particularly deep learning, provides powerful tools for extracting clinically relevant patterns from such data [8,9]. However, fair comparison and validation of these methods depend on high-quality benchmarking datasets consisting of temporally resolved patient trajectories for well-defined clinical cohorts, including reproducible preprocessing pipelines and careful control of information leakage. In the postoperative setting, this need is still largely unmet, impeding the evaluation of modern sequential models.

To support comparative ML studies on postoperative care in VR patients, the primary contribution of this work is a clinically informed and reproducible EHR dataset generation pipeline and benchmark for predictive models based on longitudinal data from the Medical Information Mart for Intensive Care (MIMIC)-IV database. Using expert-guided data curation, cohort definition, and feature selection, we construct a general-purpose dataset of VR patients, referred to as VR-general. This dataset captures perioperative physiology and laboratory measurements alongside patient history (including prior diagnoses and procedures), cardiovascular risk factors, and documented comorbidities, thereby providing a comprehensive resource that supports a wide range of downstream analytical and predictive tasks.

As an illustrative use case to demonstrate the benchmark’s utility, we predict clinically relevant postoperative complications using intensive care unit (ICU) readmission during the same hospital stay as a representative endpoint and surrogate marker for adverse short- and mid-term outcomes. From VR-general, we derive a task-specific dataset, VR-pred, by applying an information cut-off at the time of first ICU discharge and excluding potential sources of leakage, such as diagnostic codes or other variables reflecting clinical decisions or events occurring after the prediction time point. As a reproducible benchmark, we establish baseline performance by comparing (i) a Transformer that leverages temporally resolved patient trajectories with (ii) Transformer and XGBoost baselines trained on aggregated, non-temporal features in an attempt to isolate the contribution of temporal information to postoperative risk modeling.

The goal of this work is to provide a clinically grounded, high-frequency EHR benchmarking resource for postoperative care research in VR patients, together with a fully reproducible pipeline. Using an exemplary postoperative risk prediction task, we assess the added value of our temporally resolved benchmark compared with non-temporal baselines. To our knowledge, this benchmark uniquely distinguishes itself from existing MIMIC-derived datasets by combining (i) public availability (reproducible generation from MIMIC-IV with authorized access), (ii) a clinically validated feature selection and cohort definition, (iii) high-frequency, high-dimensional time-series information, and (iv) a clinically relevant postoperative endpoint. All code required to process MIMIC-IV into VR-general and VR-pred is publicly released (Code available at: https://git.iccas.de/valve-replacement-risk-stratification/dataset-pipeline-vr, accessed on 29 January 2026).

The remainder of this manuscript is organized as follows: Section 2 reviews related work on data-driven approaches for post-VR care and the role of reproducible, time-resolved EHR benchmarks. Section 3 describes construction of VR-general and the derivation of VR-pred for ICU readmission prediction, including the tokenization scheme, predictive models, and explainability methods. Section 4 summarizes cohort characteristics, evaluates ICU readmission as a postoperative risk stratifier, and reports model performance and feature attributions. Section 5 discusses implications for risk stratification and care optimization, assesses clinical plausibility of model-derived signals, and outlines methodological and clinical limitations. Section 6 concludes and highlights directions for future work.

## 2. Related Work

Preoperative risk assessment in VR surgery has traditionally relied on established risk scores such as the EuroSCORE (II) and the STS Score [10,11,12], which utilize regression techniques to guide clinical decisions. Beyond these conventional methods, a growing body of research has explored data-driven approaches to predict diverse postoperative outcomes. For instance, XGBoost models have shown improved performance in predicting reoperation after surgical aortic VR and related outcomes [13], outperforming traditional logistic regression approaches. In addition, ML models have been developed to predict a composite of postoperative complications, including stroke and renal failure, following transcatheter mitral valve repair. These models were trained on data from a multicenter cohort in Chinese hospitals that was not made publicly available for ethical reasons [14]. Several studies have investigated the prediction of one-year all-cause mortality after transcatheter aortic VR (TAVI) using XGBoost [15,16,17] and decision tree–based models [18], each leveraging different clinical databases. Furthermore, 30-day mortality after TAVI has been assessed using logistic regression and random forest approaches with data from the Netherlands Heart Registration and German Aortic Valve Registry respectively [19,20]. Other frequently reported applications of ML in VR research include the prediction of renal failure [13,21], stroke [13,14,21], and deep sternal wound infection [13,21]. While these studies highlight the growing interest in data-driven approaches for managing VR patients, their comparability and reproducibility remain limited due to non-standardized data preprocessing and restricted data access stemming from ethical constraints. Furthermore, many available datasets lack high-dimensional time-series information, forcing models to depend on summary statistics [14] or isolated single-time-point assessments [22,23,24]. As a result, many studies either lack longitudinal data altogether or employ modeling designs that disregard temporal information, preventing them from capturing the dynamics that more advanced architectures, such as Transformers, are specifically designed to leverage. Our work addresses these gaps by introducing a comprehensive pipeline for constructing an EHR dataset of VR patients that integrates high-resolution, time-dependent information—such as vital signs, medication administrations, ICU alarms, and patient histories—thereby enabling rigorous evaluation of data-driven time-series models for perioperative care.

## 3. Material and Methods

### 3.1. Baseline Dataset

**Figure 1:**
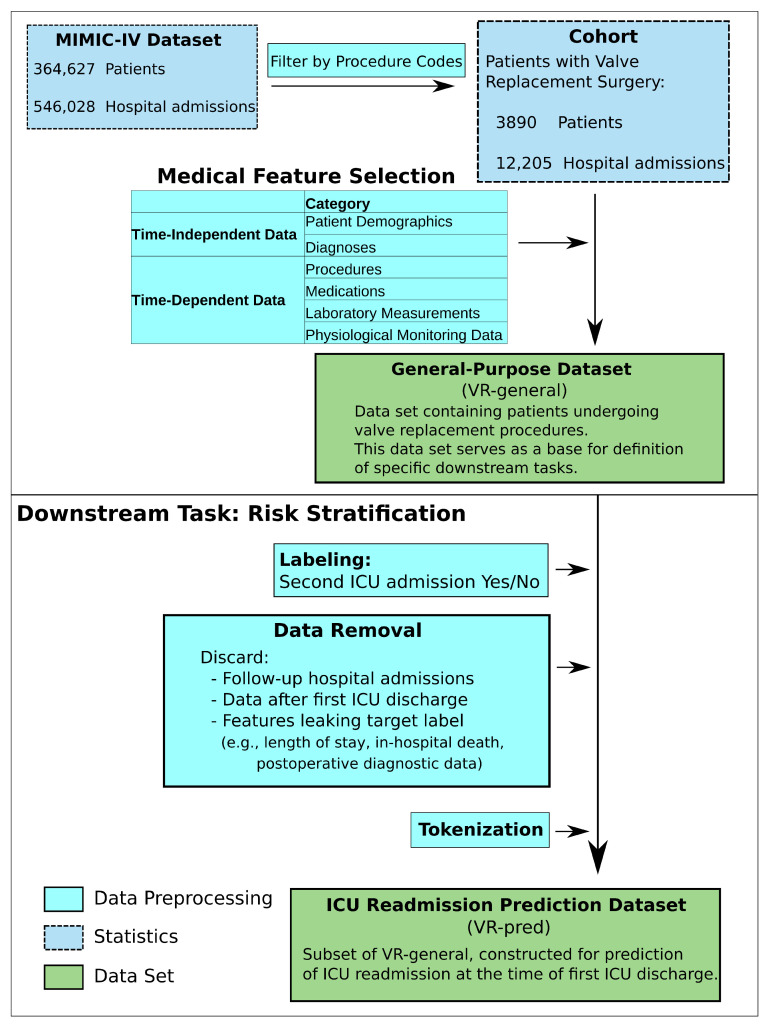
Illustration of dataset construction. **Top**: creation of the *VR-general* dataset; **Bottom**: derivation of the *VR-pred* dataset for ICU readmission prediction.

Our study is based on the Medical Information Mart for Intensive Care IV (MIMIC-IV) dataset [25,26], which contains de-identified electronic health records of patients admitted to the Beth Israel Deaconess Medical Center in Boston, Massachusetts, between 2008 and 2022. It contains ICU and anamnestic data such as patient demographics, vital signs, laboratory results, medications, diagnoses and procedures. Figure 1 gives an illustrative overview of the cohort and dataset generation process.

#### 3.1.1. Cohort Definition

For the cohort definition, we identified ICD-PCS codes corresponding to VR surgeries (for an extensive list, see Appendix A). Patients with any recorded procedure matching these ICD codes were included in the cohort. Invalid samples, like those with procedure dates outside hospital stays, were discarded. To reduce the likelihood that VR represented a secondary or incidental intervention rather than the primary reason for hospitalization, patients whose valve procedures occurred after their initial ICU admission were excluded. We restricted the cohort to VR rather than defining a broad cardiac surgery population to mitigate clinical heterogeneity. For instance, broad cardiac surgery cohorts are frequently dominated by coronary artery bypass grafting patients [27], whose primary complication risks (e.g., graft failure) differ from VR-specific risks such as paravalvular leaks. Even regarding shared adverse events, risk profiles diverge significantly between sub-cohorts. For example, the incidence of permanent pacemaker implantation varies substantially [28]. This divergence results in distinct clinically relevant prediction targets. Furthermore, a general cohort would require a substantially expanded feature set and restricting the scope to VR ensured that medically informed feature selection remained feasible. Finally, this focus enables downstream models to serve as specialized extensions to general risk tools (e.g., EuroSCORE II, STS) and ICU indices (e.g., APACHE, SOFA), rather than duplicating them. Additionally, reconstructive heart valve operations (i.e., repair without replacement) were excluded because valve repair is associated with significantly better survival and lower reoperation rates [29]. Including these lower-risk repair patients would exacerbate class imbalance for morbidity and mortality endpoints. Further, this exclusion prevents models from achieving high performance by stratifying risk solely based on procedural codes rather than analyzing patient trajectories. This choice of cohort prioritizes high internal validity and the creation of a homogeneous, high-quality benchmark over broader generalizability to more heterogeneous cardiac surgery populations.

#### 3.1.2. General-Purpose VR Dataset

We constructed the *VR-general* dataset to capture the overall clinical state of patients undergoing VR surgery through medically informed feature selection guided by the clinical–technical consensus workflow summarized in Box 1. The features of the resulting dataset are organized into the following aspects: **Patient history** included previous diagnoses and procedures, as well as interventions and medications administered during the VR hospitalization. **Risk factors for cardiovascular disease** were incorporated, including modifiable factors such as hypercholesterolemia (reflected by laboratory parameters LDL, HDL, and triglycerides), hypertension, tobacco use, diabetes mellitus (reflected by blood glucose and HbA1c), and adiposity [30], as well as non-modifiable factors such as age and gender [31]. We considered **comorbidities** given their known impact on outcomes after aortic VR [32]. A relevant subset—including anemia, atrial fibrillation, chronic kidney disease, chronic obstructive pulmonary disease, and renal failure—was identified by drawing parallels with related cardiac conditions (e.g., congestive heart failure) [33] and surgical procedures (e.g., coronary artery bypass grafting) [34]. Accordingly, we incorporated parameters reflecting the involved organ systems, including pulmonary, renal, hepatic, and hematologic function. To represent the patient’s **postoperative state**, physiological parameters (e.g., body temperature, heart rate and coagulation status) were considered. To indicate **postoperative complications**, we included features related to infections (e.g., white blood cell count and details of antibiotic use [35]) and mechanical complications as defined in Table A3 (e.g., ejection fraction [36] and hyperlipidemia [37]). For thromboembolic events, we integrated blood coagulation values such as INR, PT, aPTT, fibrinogen, and platelets [38]. Further, we integrated established risk factors of postoperative complications after VR (e.g., body surface area for periprosthetic regurgitation and paravalvular leaks [39,40]). A more detailed overview of the manually selected features for each category is provided in Appendix B.

Box 1 **Summary of the iterative feature selection refinement process through clinical–technical collaboration.**

Clinical-Technical Consensus Workflow for Feature Selection
**Step 1. Clinical Domain Scoping:** A physician identified clinical parameters required to represent patient status and outcomes, guided by established cardiac surgery guidelines and relevant literature.
**Step 2. Mapping to MIMIC-IV Schema:** These parameters were mapped to the respective concepts within the MIMIC-IV database.
**Step 3. Iterative Consensus Review:** Parameters unavailable in the database were either substituted with clinically valid proxies or excluded. Additionally, the timing of measurements was medically reviewed to ensure clinical relevance.

### 3.2. Use Case: Postoperative Complication Risk Prognosis

To illustrate the potential of the *VR-general* dataset as a benchmark for predictive modeling, we evaluate its utility in prognostic stratification of patients by their risk of postoperative complications.

#### 3.2.1. Label Definition

MIMIC-IV does not provide a direct indicator of postoperative complication risk following VR surgery. Although mortality constitutes a straightforward prediction target, its causal attribution to VR surgery cannot be ensured in MIMIC-IV, and non-lethal postoperative complications are not systematically captured. Furthermore, post-discharge mortality tracking is limited to one year, constraining long-term outcome evaluation. Another potential strategy for creating morbidity-inclusive labels would involve using diagnostic codes, possibly in combination with mortality data. However, in MIMIC-IV, diagnostic codes are recorded at the admission level without temporal annotations. This poses two challenges: incorporating codes from the same admission as the VR surgery risks label leakage, whereas restricting the analysis to subsequent admissions may miss early complications (e.g., mechanical dysfunction, atrial fibrillation, or postoperative infections) that were managed during the initial stay. Consequently, defining reliable prediction targets based on diagnostic codes is not feasible for our use case in MIMIC-IV. Given these limitations, a surrogate endpoint is required to approximate postoperative risk. In medical research, when direct or ideal clinical outcomes are difficult to obtain, the use of well-justified proxy targets or surrogate endpoints is a common and pragmatic strategy [41,42]. In this context, we may consider ICU readmission a suitable prediction target, as it reflects acute patient deterioration (e.g., cardiovascular instability or heart failure [43]) and thus serves as a meaningful proxy for postoperative risk. To evaluate its validity as such, we later examine its association with several common postoperative complications. The risk assessment is performed at the time of first ICU discharge, which allows adequate data collection and represents a clinically meaningful opportunity to adjust postoperative management according to the predicted risk.

#### 3.2.2. Predictive Model Specifications

**Figure 2:**
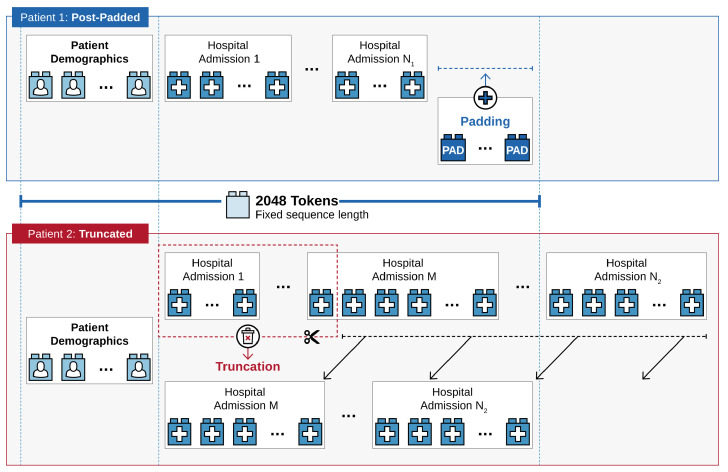
Schematic illustration of the tokenization scheme. Token sequences begin with demographics, followed by chronological hospital admission blocks containing time-independent and time-dependent events. To align with the 2048-token limit, shorter sequences are post-padded, whereas the earliest admission-specific data is truncated from longer sequences.

To evaluate the added value of temporal information in postoperative risk prediction, we compare two modeling paradigms for ICU readmission prediction at the time of first ICU discharge: a self-attention-based Transformer model capable of leveraging sequential EHR data against a XGBoost baseline trained on aggregated, non-temporal features. *Self-attention-based Transformer models* have demonstrated exceptional performance across various domains [44], including processing of longitudinal EHR data [45,46]. We extend the tokenization scheme introduced by Li et al. [45] to convert patient data into token sequences. For this purpose, we order patient data by hospital admission and arrange time-dependent data chronologically. While categorical features are tokenized directly, continuous variables are discretized using quantile-based binning. For example, heart rate values around 80 bpm typically fall into a mid-quantile bin that corresponds to a clinically normal range (i.e., normocardic-range), whereas values around 130 bpm fall into an upper-quantile bin that corresponds to a tachycardic range (i.e., tachycardic-range). This tokenization emphasizes clinically salient state changes (i.e., transitions between ranges) while remaining robust to minor within-range variability. A discussion of the quantile-based discretization strategy and its alternatives is provided in Appendix C. Under this tokenization scheme, missing values are handled implicitly: if a measurement or clinical event is not observed, the corresponding token simply does not appear in the patient’s sequence. We analyzed the distribution of token sequence lengths and observed a long-tail distribution. To maintain computational feasibility, sequence lengths were capped to 2048 tokens. Sequences shorter than the maximum length were post-padded with a designated padding token. Sequences exceeding the limit were truncated by removing the earliest time-dependent events. This strategy explicitly preserved static features such as patient’s demographics, long-term events like historical diagnoses, and the most recent clinical trajectory. To handle varying sequence lengths within the model, we employed a binary attention mask to prevent the model from attending to padding tokens. A schematic illustration of the tokenization scheme is shown in Figure 2, and a more detailed description, including an example, is provided in Appendix C. Token sequences are converted into numerical representations through learned embeddings. To preserve temporal relationships between events, sinusoidal positional embeddings [44] are added to encode time information. The final input to the Transformer model is obtained by summing the learned token embeddings with their corresponding temporal (sinusoidal) embeddings. We use a multi-layer, decoder-only Transformer architecture [47]. Model hyperparameters were selected through a grid-based search including embedding size, number of layers, number of attention heads, and the dimensionality of the feed-forward layers. We defined the hyperparameter search space guided by prior studies on Transformer-based EHR sequence modeling: embedding dimensions between 128 and 768 [48,49], depths of four to six layers [45,50], and feed-forward network dimensionalities from 64 to 256 [50,51]. The final configuration employs an embedding dimension of 768 and comprises 6 layers, each with six attention heads, a fully connected feed-forward network of dimension 256, and layer normalization. The Transformer was trained using the Adam optimizer [52] with a learning rate of $3\times 10^{-5}$. The model was optimized using a binary cross-entropy loss function. Training was performed with a batch size of 32, a dropout rate of 0.1 within the Transformer layers, and early stopping with a patience of five epochs.

As a complementary baseline representing traditional ML, we employ an *XGBoost* classifier. XGBoost is a well-established and high-performing algorithm widely used in clinical prediction tasks [53,54,55]. Unlike the Transformer, this model operates on aggregated, non-temporal features and does not explicitly capture sequential dependencies. Its input is obtained from the tokenized dataset by counting the frequency of each token across a patient’s record, yielding a fixed-length vector that serves as a static summary of the EHR data. In this representation, missing data correspond to a zero count for the respective tokens, preserving the distinction between the absence of a measurement and tokens that explicitly encode a measured value of zero. The XGBoost classifier was trained using a binary logistic objective, a learning rate of 0.3, a maximum tree depth of 6, and 100 boosting rounds. $L_{1}$ and $L_{2}$ regularization parameters were set to α=0 and λ=1, respectively. Row and feature subsampling rates were both fixed at 1.0, using the entire dataset in each boosting iteration. This configuration was determined through hyperparameter tuning over a search space derived from prior benchmarks applying XGBoost to EHR data. The specific parameter ranges were: learning rate 0.1–0.35 [56,57], maximum tree depth 3–10 [58,59], number of boosting rounds 80–500 [53,56], α 0–0.1 [59,60], and λ 0–1 [57,60]. As a second baseline, we included a Transformer model trained on the same aggregated, non-temporal features. Together, these baselines allow us to quantify the incremental predictive value of explicitly modeling temporal dynamics for prognostic tasks.

All the models were evaluated using cross-validation, applying an 8:1:1 split within each fold to partition the data into training, validation, and test subsets. To prevent information leakage, this partitioning was performed at the patient level, ensuring that all the admissions belonging to the same patient remained within the same subset. All the models were trained on a workstation equipped with an NVIDIA GeForce RTX 3090 GPU with 24 GB VRAM. The implementation utilized Python 3.13.5 and PyTorch 2.7.0.

#### 3.2.3. Benchmark VR Dataset

To derive the *VR-pred* dataset for the specific task of predicting ICU readmission, we modified the *VR-general* dataset to ensure information-leakage control by restricting data to information available at the time of prediction (the first ICU discharge).

1. **Future Hospitalizations:** We discarded all data associated with hospital admissions that occurred after VR hospitalization.
2. **Time-Series Cut-off:** For the VR hospitalization, all time-dependent data (i.e., laboratory measurements, medications, physiological monitoring data, and procedures) with timestamps occurring after the first ICU discharge were removed. Since procedures in MIMIC-IV are recorded at a daily resolution only, all procedures dated on the day of ICU discharge were also excluded.
3. **Untimed Data:** We manually reviewed patient demographics and admission metadata, explicitly excluding total length of hospital stay, discharge time, and in-hospital mortality flags, as these features provide strong indications regarding the prediction target.
4. **Diagnostic Codes:** Diagnostic codes are recorded only at hospital-admission granularity and lack precise timestamps. To avoid leaking postoperative information, we, therefore, excluded all diagnostic codes assigned during the VR hospitalization. Only diagnoses from prior hospital admissions were retained to characterize pre-existing comorbidities.

An overview of the dataset generation process is provided in Figure 1.

#### 3.2.4. Feature Relevance and Attribution Methods

To evaluate a measure of model- and time-independent feature relevance for predicting ICU readmission, we performed a pairwise correlation analysis. Using aggregated, time-independent feature statistics, we quantified the relationship between token occurrence frequency and ICU readmission with the $\phi_{k}$ correlation coefficient, which captures non-linear dependencies [61]. Since pairwise correlation measures do not account for the complex feature intercorrelations exploited by ML algorithms, we employ feature attribution methods for the considered predictive models: *Integrated Gradients* [62] and *SHapley Additive exPlanations (SHAP)* [63]. Integrated gradients attribute a model’s prediction to input features by integrating output gradients along a baseline-to-input path, yielding attributions that sum to the prediction difference between input and baseline. This technique addresses limitations of other methods by satisfying two fundamental axioms: sensitivity, which ensures features altering a prediction receive non-zero attribution, and implementation invariance, guaranteeing identical attributions for functionally equivalent networks regardless of their internal structure. SHAP estimates each input feature’s contribution to the model’s output leveraging Shapley values originating from cooperative game theory. Shapley values attribute a model’s prediction to its input features, calculating each feature’s average marginal contribution (i.e., the expected change in model output when the feature is added).

## 4. Results

In this section, we describe the VR surgery cohort derived from MIMIC-IV, which we subsequently use as the basis for our example downstream predictive study of postoperative complication risk. To validate our surrogate prediction target (second ICU admission), we analyze correlations between secondary ICU admissions and key postoperative outcomes, including mortality and the most prevalent complications. We then compare predictive performance between the Transformer time-series approach and the non-temporal baselines, and study feature importance with both methods.

### 4.1. VR-General Cohort

The *VR-general* cohort represents a comprehensive, high-resolution dataset of patients who underwent VR surgery, derived from systematic preprocessing of the MIMIC-IV database. It comprises a total of 3890 patients, including 3224 with aortic, 613 with mitral, 50 with pulmonary, and 3 with tricuspid VRs. An overview of key demographic and clinical characteristics is provided in Table 1. The dataset integrates 6313 distinct features organized into six clinically meaningful categories (see Figure 1): patient demographics (5), diagnoses (3901), procedures (1650), medications (652), laboratory measurements (72), and physiological monitoring data (33). This range of information captures the full perioperative context of VR surgery, ranging from chronic comorbidities and intra-hospital interventions to detailed laboratory and physiological measurements and enabling multimodal, time-resolved analyses of patient trajectories.

### 4.2. Case Study: Postoperative Complication Risk Prognosis

#### 4.2.1. ICU Readmission as Surrogate Target for Postoperative Complication Risk

**Figure 3:**
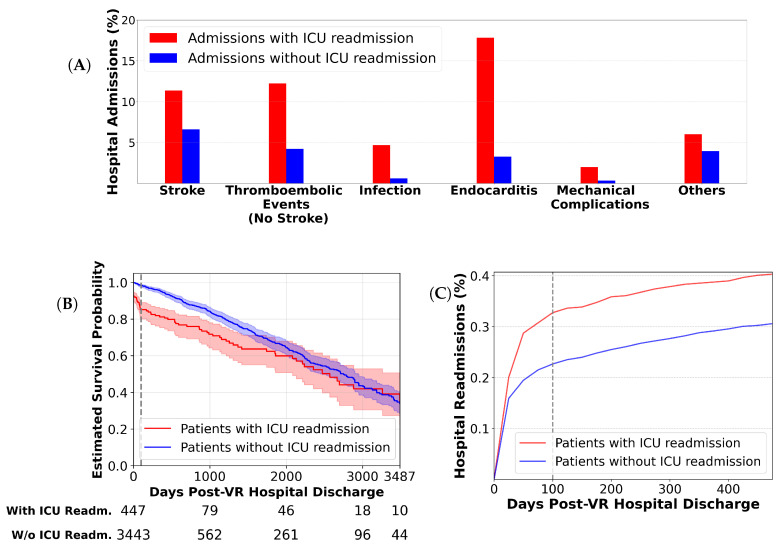
Valve replacement cohort stratification by ICU readmission: (**A**) Occurrences of in-hospital postoperative complications (for included ICD-CM diagnostic codes, see Appendix D); (**B**) Kaplan–Meier survival analysis including 95% confidence intervals and number-at-risk table, x axis is truncated when fewer than 10 patients remain at risk in the ICU-readmission cohort; (**C**) hospital readmission rate after hospital discharge.

Within our VR-general cohort, 3443 (88.5%) patients had exactly one ICU admission during their hospital stay (predictive target class 0), and 447 (11.5%) patients had one or more secondary ICU admissions (target class 1). In-hospital mortality during the VR hospitalization was limited exclusively to the readmission cohort (32 patients, 0.8% of total): 26 patients died during the secondary ICU stay, and 6 died on the ward following discharge. Hospitalization duration deviated significantly between the two main groups: patients without ICU readmission stayed on average 9.0±6.3 (μ±σ) days, compared to 21.4±16.1 days for those readmitted to the ICU. In our cohort, ICU readmission clearly stratified patient risk with respect to common in-hospital postoperative complications, with higher prevalence observed for general surgical risks such as stroke, thrombosis, and infections, as well as VR–specific complications including endocarditis and mechanical prosthesis dysfunction (Figure 3A). Extending the analysis beyond the index hospitalization, we observed that ICU readmission also stratified patient risk within 100 days post-discharge. Patients in the ICU-readmission cohort exhibited higher rates of hospital readmission (Figure 3C), indicating an increased susceptibility to mid-term complications. For the Kaplan–Meier survival analysis, we included all in-hospital deaths during the VR hospitalization and any subsequent readmissions, as well as extra-hospital deaths recorded within MIMIC-IV’s one-year follow-up period. The analysis revealed a markedly reduced survival probability for patients in the ICU-readmission cohort, as illustrated in Figure 3B (Right-censored observations, i.e., vertical ticks, represent patients discharged alive who remained event-free throughout the follow-up period).

#### 4.2.2. Predictive Model Performance

We evaluated the performance of both Transformer and XGBoost models in predicting ICU readmission using 10-fold cross-validation on the *VR-pred* dataset. To disentangle the effects of temporal information from those of model architecture, the Transformer was trained on two input variants: (i) sequential token data from *VR-pred*, and (ii) aggregated feature statistics identical to those used for XGBoost. As summarized in Table 2, we report AUROC and AUPRC. In our experiments, the sequential Transformer achieved the highest mean cross-validation performance for both metrics. Compared with the non-sequential variant, the sequential model improved AUROC by 0.048 (5.8%) and AUPRC by 0.126 (22.5%). Relative to the XGBoost baseline, SeqT achieved higher performance, with AUROC increasing by 0.044 (5.3%) and AUPRC by 0.061 (9.8%). In the comparison of models restricted to aggregated (static) feature statistics, XGBoost achieved higher scores than the non-sequential Transformer. To investigate the statistical significance of the differences in predictive performance, we applied corrected resampled *t*-tests [64] on the pairwise metric differences using five independent 10-fold cross-validation runs. Because our primary hypothesis was directional, namely that preserving temporal information yields higher discrimination than non-temporal feature aggregation, we report one-sided corrected resampled *t*-test *p*-values for the SeqT vs. NonSeqT/XGBoost comparisons. For a detailed description of the approach we refer to Appendix F. The results of the *t*-tests are summarized in Table 3. The comparison between SeqT and NonSeqT showed a statistically significant performance advantage of SeqT in both AUPRC and AUROC, consistent with the hypothesis that exploiting temporal order improves discrimination. Comparisons between SeqT and XGBoost also favored SeqT, albeit with weaker statistical evidence. XGBoost, in turn, significantly outperformed NonSeqT in AUPRC but not in AUROC. Taken together, these results support the interpretation that the performance gains of the Transformer are primarily attributable to temporal information rather than architectural differences alone.

#### 4.2.3. Feature Importance

To understand the key drivers behind the prediction of a second ICU admission and to assess the clinical plausibility of our models, we conducted a comprehensive feature importance analysis. This investigation employed three distinct methodologies to provide a multifaceted view of feature relevance. As model-independent assessment we used the $\phi_{k}$ correlation coefficient [61]. Second, for explainability of the Transformer, we assessed feature attributions calculated using the *Integrated Gradients* method [62]. Third, for XGBoost trained on aggregated feature statistics, we employed SHapley Additive exPlanations (SHAP) [63]. Table 4 presents the importances of the different feature categories for each method, showing the relative mean feature importance (i.e., “How important is a typical feature from this category?”), the relative cumulated category importance (“How much contributes the feature class overall?”), and the size-normalized category importance (“Does this category contribute more/less importance than expected given how many features it contains?”). While Table 4 allows a high-level overview, a more granular inspection of the three most influential individual features identified within each respective category is available in Table A4, Table A5 and Table A6 in the Appendix E.

## 5. Discussion

In this section, we discuss the contributions and implications of our work, beginning with the *VR-general* dataset itself. We first elaborate on its design, clinical validity, and intended utility as a reproducible, high-resolution time-series benchmark for comparative AI studies. We then transition to a detailed analysis of the exemplary downstream task of predicting postoperative complication risk. This analysis begins with an examination of our chosen surrogate target, ICU readmission, and its suitability for postoperative risk stratification. We then analyze the predictive performances achieved by the models, focusing on the distinction between the time-series Transformer and the static, summary-statistics-based XGBoost baseline. Following this, we provide a medical assessment of the most important features identified by the attribution methods. Finally, we discuss the broader implications of these findings for both clinical decision support and future research.

### 5.1. A Clinically Valid, Reproducible Time-Series Cohort

As major contribution, we introduced the *VR-general* dataset and an end-to-end, code-released pipeline that transforms raw MIMIC-IV data relevant to VR surgery into clinically anchored patient trajectories. This resource is built from a transparent cohort definition, removes invalid samples, and organizes features into a schema whose time windows are aligned with meaningful peri-operative events across history, diagnoses/procedures, labs, medications, and physiological monitoring. Together, these design choices provide a standardized foundation for fair comparisons between predictive time-series models lowering the barrier to robust benchmarking of data-driven models for perioperative care.

### 5.2. ICU Readmission as Postoperative Risk Stratifier

ICU readmission emerged as a meaningful stratifier of postoperative complication risk within our VR cohort. Patients who required a second ICU stay showed markedly higher in-hospital mortality and longer hospitalizations, whereas single-ICU patients exhibited lengths of stay comparable to published cardiac surgery averages [65]. Because postoperative complications are key determinants of prolonged recovery [65], we examined the occurrence of specific in-hospital complications, including common general postsurgical and cardiac-specific complications [1] (see Figure 3A). Across all complication types, the ICU-readmission cohort consistently exhibited higher incidences. The stroke rate of 11.4% in this group exceeded the 1–10% range typically reported after VR [66,67,68], while the 6.6% rate among single-ICU patients aligned with literature values. While literature on VR thromboembolic events primarily reports stroke and venous thromboembolisms with incidences of 1.3–3.2% [69,70], an incidence of 2.5% for arterial thromboembolisms excluding stroke within 30 days after cardiac surgery was reported [71]. Although methodological differences preclude direct comparison, elevated thromboembolic event rates (12.3% vs. 4.2%) consistently signal increased complication burden. Infections and endocarditis followed the same pattern: infection rates (4.7% vs. 0.6%) surpassed typical postoperative infection rates of 1–2% [72], and endocarditis occurred fivefold more often in the ICU-readmission group (17.8% vs. 3.3%), the latter closely matching published baseline rates [73,74]. The broad spectrum of mechanical complications makes direct comparison to existing literature challenging. However, while complications like pannus ingrowth and structural valve dysfunction typically occur in the long term and not during VR hospitalization, paravalvular leaks are often identified at earlier postoperative stages, with reported rates ranging from 0.6% to 12% [75,76]. While the rates for both cohorts fall within this range, the incidences for the ICU-readmission cohort are markedly higher (2% vs. 0.3%). Notably, all in-hospital deaths (0.8% of the entire cohort) occurred exclusively among ICU-readmitted patients. Collectively, these findings support ICU readmission as an indicator of elevated short-term postoperative morbidity and mortality. Survival analyses extended this relationship beyond index hospitalization discharge. Kaplan–Meier curves revealed substantially reduced long-term survival among ICU-readmitted patients (Figure 3B), with two-year survival rates falling within the 69–90% range reported in earlier studies [77,78]. Furthermore, hospital readmission within 100 days post-discharge was 50% higher in the ICU-readmission group, while longer-term readmission rates converged between cohorts(Figure 3C). Together, these observations establish ICU readmission as a clinically robust and temporally stable surrogate for both immediate and mid-term postoperative risk after VR surgery within the scope of the analyzed MIMIC-IV cohort. Limitations of the proxy target regarding institutional variability in ICU admission policies and potential misclassification of specific adverse events are discussed later in Section 5.5.

### 5.3. Predictive Performance in Postoperative ICU Readmission Classification

Predictive performance (Table 2 and Table 3) supports the feasibility of estimating ICU readmission risk at the time of ICU discharge, consistent with clinical practice in assessing pre-transfer stability and early deterioration risk. Accordingly, ML models have the potential to complement established general-purpose scores such as DRS [79] and SWIFT [80] by providing VR-specific postoperative risk stratification. Notably, the sequential Transformer trained on temporally resolved trajectories achieved the best overall performance. The corrected resampled *t*-test supports a statistically significant improvement over the aggregated-feature Transformer (for both AUROC and AUPRC), whereas SeqT’s numerically higher scores than XGBoost did not reach statistical significance in either metric. In the aggregated-feature setting, XGBoost was comparable to the non-sequential Transformer in AUROC and showed a statistically significant advantage in AUPRC, consistent with the strong performance of tree-based methods on tabular summaries. Together, these findings indicate that the sequential Transformer’s advantage primarily arises from exploiting longitudinal time-series information and highlight the value of temporally structured datasets for postoperative outcome prediction. In contrast, aggregating trajectories into static representations discards clinically important temporal context such as trend direction (patient state improving vs. worsening), recency (whether abnormalities occur early or shortly before ICU discharge), persistence (sustained vs. transient episodes), and event ordering (e.g., escalation of support before vs. after deterioration). Consequently, two patients can exhibit identical aggregated features, yet represent fundamentally different courses (such as gradual stabilization versus late acute decompensation).

### 5.4. Feature Importance: Method Caveats and Clinical Plausibility

We summarize aggregated category-level feature importances for ICU readmission in Table 4. Note that each method captures a different facet of “importance” and magnitudes are not directly comparable across methods.

*Methodological perspective.* We used the $\phi_{k}$ coefficient as a pairwise association measure on the aggregated feature space (token occurrence counts). $\phi_{k}$ can capture non-linear relationships and is independent of any downstream predictive model. As a purely pairwise statistic, however, it cannot represent higher-order dependencies across multiple features and is susceptible to confounding beyond the two-variable scope. At the same time, this pairwise perspective is not affected by multicollinearity artifacts. In contrast, *SHAP* and *Integrated Gradients* (IG) are model-specific attribution methods: they explain a trained model’s predictions and can reflect complex interactions learned by that model. Both are sensitive to multicollinearity, such that importance may be distributed across correlated features without uniquely identifying the underlying causal driver. In our experiments, SHAP is applied to XGBoost trained on aggregated (non-temporal) features, whereas IG is applied to the sequential Transformer operating on temporally resolved token sequences. Consequently, only IG can attribute importance to time-localized information.

*Category-level trends.* We analyzed feature importance for ICU readmission prediction using a model-agnostic, pairwise association measure ($\phi_{k}$) and two model-based attribution methods: Integrated Gradients (IG) for the sequential Transformer and SHAP for XGBoost trained on aggregated summary features. The $\phi_{k}$ analysis suggests that all feature categories contain predictive signal, most notably laboratory markers. However, because $\phi_{k}$ quantifies pairwise associations at the single-feature level, category totals can be inflated by redundancy within a category and should therefore be interpreted cautiously. Across $\phi_{k}$, SHAP, and IG, laboratory features, which are temporal high-resolution but irregularly sampled, emerge as the dominant drivers of prediction and account for the largest share of cumulative category importance. This indicates that acute physiologic derangement is the primary determinant of second ICU admission risk. Clinically, this is plausible: laboratory measurements often reflect organ dysfunction and postoperative complications and can change within hours. At the same time, the strong SHAP mass on laboratory summaries suggests that aggregated statistics already capture a substantial fraction of this signal, likely because extreme or threshold-crossing values are highly informative even without explicit temporal context. In contrast, physiological monitoring, high-frequency and cyclic, shows a different pattern. While $\phi_{k}$ indicates that monitoring variables are associated with the outcome in the aggregated representation, XGBoost assigns them virtually no importance, whereas the sequential Transformer attributes substantially more weight to this category. This divergence is consistent with the idea that monitoring signals become predictive primarily through their temporal dynamics (e.g., trends, variability, abrupt changes), which are flattened by aggregation and therefore difficult for XGBoost to exploit. Demographics are static at the patient level and exhibit consistently high per-feature importance across methods, but contribute little cumulative category importance due to the small number of demographic variables. Historic diagnoses show non-trivial pairwise association under $\phi_{k}$, yet receive little weight under SHAP and only minor weight under IG, suggesting that their signal is comparatively weak once acute physiologic information is available, or that it is partly absorbed by other correlated covariates. Procedures, recorded at daily resolution per hospital admission, are strongly associated with the target and are correspondingly used by both XGBoost and the Transformer. A similar pattern holds for medications, which are recorded at higher frequency and contribute moderately under both model-based methods. Overall, the Transformer distributes importance more broadly across categories, whereas XGBoost concentrates attribution primarily on laboratory-derived summaries. Taken together, these results indicate that all top-level feature categories are relevant for ICU readmission prediction, but that the sequential Transformer additionally leverages information that is expressed through temporal structure—most clearly in physiological monitoring and, to a lesser extent, temporally anchored clinical history—supporting the value of modeling these data in their sequential context rather than relying solely on aggregated summaries.

*Clinical plausibility of selected features.* Below, we discuss representative high-ranking features per category and why their importance is medically credible. The specific features discussed, along with their detailed importance scores, are provided in Appendix E.

**Procedures:** Among the most important features identified in this category were indicators of prolonged intubation and re-intubation. Following a complication-free operation, patients are typically extubated within a few hours, with time-to-extubation having been proposed as a predictor for postoperative recovery in previous studies [81,82]. Further, studies have consistently shown that prolonged mechanical ventilation is a significant predictor of increased ICU readmission rates [83,84], which directly validates the high importance assigned to mechanical ventilation exceeding 96 h. The occurrence of prolonged ventilation further offers a plausible explanation for the wide variance in hospital length of stay reported in Table 1 (median 5.0 vs. 95th percentile 20.5 days), suggesting respiratory complications drive these prolonged trajectories. Furthermore, endotracheal airway insertion (defined herein as re-intubation, distinct from the initial surgical intubation, which is not captured within the data) and the initiation of nasogastric or percutaneous endoscopic gastrostomy feeding directly indicate severe physiological compromise (such as respiratory failure, compromised airway protection, or impairment of deglutition) and have been associated with adverse outcomes including increased mortality or pneumonia [85,86]. Consequently, these procedures are indicators of a complication-prone surgical or early postoperative course, signaling greater illness severity and complexity, which in turn increases the risk of ICU readmission [87].

**Diagnoses:** The most important diagnoses reflect significant pre-operative cardiac damage (e.g., ischemic cardiomyopathy) or an increased risk thereof (e.g., hypertensive chronic kidney disease [88]), with the latter aligning with the high prevalence of renal comorbidities reported in Table 1. This damage is associated with an elevated risk of various postoperative complications, finally leading to ICU readmission [89,90,91,92]. Additionally, severe sepsis with septic shock increases the risk of sepsis recurrence and often induces cardiac damage, making it a significant indicator of ICU readmission [93,94]. The emphasis on sepsis is further justified by the cohort distribution shown in Table 1, where infectious complications (6% endocarditis, 3% general infection) represent a major class of postoperative adverse events.

**Laboratory:** The most important features in this category were arterial blood gas markers. ICU readmissions are predominantly caused by respiratory failure (59%), cardiovascular instability (25%), and renal failure (6.5%) [43]. These complications are often preceded by physiological derangements detectable in blood gas analysis, specifically through alterations in pH, $pO_{2}$, and base excess. Further, erythrocyte concentrations can indicate anemia, while glucose concentration and the administration of insulin glargine may signal diabetes mellitus [95]; both conditions are well-known risk factors for individuals with heart disease [96,97,98,99], and represent highly prevalent comorbidities in our study population (Anemia: 28%, Diabetes Mellitus: 20%; see Table 1).

**Medication:** Analysis of medications reveals docusate sodium, a stool softener, as having high predictive relevance. Although there are no evidence-based guidelines for the use of stool softeners after cardiac surgery, they are most likely used to prevent straining, which was identified as a cause of significant hemodynamic changes and potential cardiac arrest in previous studies [100]. In the absence of guidelines for the routine use of stool softeners, it can be hypothesised that their high predictive importance reflects their selective prophylactic use in patients with severe pre-existing cardiac conditions (e.g., previous open-heart surgery) who are at a higher risk of postoperative complications [101,102,103]. Antibiotics, specifically cefazolin and vancomycin, are also among the important medications. Cefazolin is routinely administered in the US for perioperative and postoperative infection prophylaxis, while vancomycin is used in exceptional circumstances. While official guidelines lack specific details on prolonged antibiotic prophylaxis administration for high-risk patients [104], those who have already received preoperative antibiotics (e.g., for endocarditis, a common reason for heart VR [105]) might undergo such extended treatment. Consequently, prolonged antibiotic administration could indicate patients at increased risk of decompensation. The continuous administration of propofol indicates a requirement for sustained sedation. This is typically necessary to facilitate mechanical ventilation or manage physiological stress, thereby serving as an example of feature collinearity and indicating increased illness severity [106].

**Physiological monitoring:** In physiological monitoring, diastolic pulmonary artery pressure and systolic arterial blood pressure show high importance. Pulmonary artery pressure directly reflects the hemodynamic load on the right ventricle, thus holding prognostic relevance [107]. Similarly, arterial systolic blood pressure provides insight into circulatory status and has been previously identified as a predictor of ICU readmission [108].

**Demographics:** Patient age, a well-established general risk factor reflecting cumulative comorbidity and diminished physiological reserve, emerges in our analysis as a relevant indicator of ICU readmission, consistent with previous research [109,110].

In summary, the most predictive features predominantly reflect cardiac-specific and general risk factors associated with an increased likelihood of ICU readmission.

### 5.5. Study Limitations

While ICU readmission was proposed as a suitable surrogate for postoperative risk, the results shown in Figure 3A warrant cautious interpretation. Although our analysis incorporates hospital readmissions and mortality events, the underlying causes of these outcomes remain unclear, preventing direct attribution to VR and restricting our findings to statistical associations. Moreover, long-term (>1 year) extra-hospital mortality data are not available. The absence of timestamps for diagnostic codes within a hospitalization in the MIMIC-IV dataset allows for potential inversion of causal relationships. For example, the high incidence of endocarditis in the ICU-readmission cohort may reflect pre-existing endocarditis leading to valve damage, VR, and subsequent ICU readmission. Furthermore, a fundamental limitation in the use of EHR data is the ambiguity between clinical events that did not occur and those that were simply not documented. Additionally, ICU readmission is a noisy indicator for postoperative complications due to institutional workflow variations and local care pathways. For instance, many U.S. hospitals manage stroke patients in dedicated stroke units rather than ICUs [111]. Consequently, only the most severe stroke cases requiring intensive interventions, such as intracranial pressure management or hypothermia therapy, result in ICU readmission, potentially leading to underestimation of stroke-related postoperative risk. Also the presence of Intermediate Care Units (IMCUs) can act as a buffer, allowing patients with moderate deterioration to be treated without formal ICU readmission [112]. Furthermore, readmission decisions are often dynamic, influenced by bed scarcity and triage thresholds during periods of high occupancy [113]. Similarly, this surrogate endpoint theoretically risks misclassifying ward-based fatalities occurring without readmission as non-events, although no such cases were observed in our cohort.

Next, the temporal granularity in MIMIC-IV differs across modalities. While physiologic monitoring and laboratory variables are time-stamped at high frequency, procedures are recorded only at daily resolution and diagnoses are documented at the hospital-admission level. Consequently, neither procedures nor the timing of diagnoses can be reliably aligned at sub-daily scale relative to high-frequency events. To mitigate any risk of label leakage arising from this mismatch, we conservatively excluded all diagnoses from the index hospital stay and all procedures dated on the ICU discharge day. This safeguards the integrity of the cut-off, at the cost of potentially increased under-documentation of clinically relevant events. Additionally, residual within-day misordering may influence token-level feature attribution for the Transformer. Because our tokenization encodes absence implicitly as the non-occurrence of an event token (or a zero count in aggregated baselines), models may exploit non-recording patterns that reflect clinical workflows or under-documentation rather than true clinical absence, particularly for sparsely documented variables. This is a general limitation of real-world EHR data.

Finally, a significant limitation regarding the dataset’s broader utility is its single-center origin. The cohort is derived exclusively from the MIMIC-IV database, reflecting the clinical practices, patient demographics, and administrative protocols of a single academic medical center in the United States. Patient populations, surgical techniques, perioperative care standards, and the criteria for ICU readmission can vary substantially between institutions and geographic regions [87,114,115]. Robust, widely deployable models will therefore require external validation on multi-institutional cohorts to ensure that learned associations do not primarily capture site-specific patterns. However, our primary objective was to provide a standardized resource for research and model comparison, rather than to present a system intended for clinical deployment. Nevertheless, we grounded our feature set in established clinical guidelines and prior multi-center evidence to support clinical relevance beyond the source institution. Accordingly, the dataset provides a useful foundation for comparative methodological research and predictive benchmarking.

## 6. Conclusions

We presented a clinically curated and fully reproducible time-resolved EHR dataset for patients undergoing valve replacement surgery designed to facilitate transparent benchmarking of data-driven predictive approaches in perioperative care. We derived the dataset from the MIMIC-IV database by medically informed cohort definition and feature selection. It integrates longitudinal, high-resolution time-series information across laboratory, procedural, medication, and physiological domains. All code necessary for generating the dataset is publicly released to ensure full reproducibility and foster verifiable, reusable data science research. As a multidisciplinary resource, our pipeline serves several distinct roles. Medical educators can utilize the dataset to demonstrate the integration of EHR data structures with machine learning-based clinical decision support and AI interpretability. Simultaneously, methodologists can leverage the benchmark for the standardized comparison of sequential architectures, while clinical researchers can develop specialized tools tailored to specific clinical challenges within postoperative valve replacement care.

In order to show the utility of the benchmark dataset, we demonstrated the prediction of postoperative complication risk as an exemplary predictive downstream task. For this purpose, we identified ICU readmission following valve replacement surgery as a suitable proxy target, given its association with increased mortality and common postoperative morbidities short and mid-term. In this exemplary task, the sequential Transformer achieved the highest mean performance and showed a statistically significant improvement over the non-sequential Transformer trained on aggregated features. Compared with XGBoost, performance was numerically higher but did not reach statistical significance under the corrected resampled *t*-test. These findings highlight the clinical relevance of integrating temporal dependencies in postoperative risk assessment. Identifying high-risk patients at ICU discharge could guide perioperative management through intensified monitoring or preventive measures, ultimately reducing complications and improving outcomes. Furthermore, predicting ICU readmission can enhance ICU scheduling systems via improved prognoses of emergency admissions [116].

In our explainability studies, the most predictive features identified by feature attribution analyses reflected well-established cardiac and systemic risk factors. This supports both the dataset’s clinical validity and the interpretability of the proposed modeling approach.

Future work should leverage the presented dataset to assess the benefits of integrating time-series information into additional perioperative prediction tasks through similar comparative ML studies. Potential applications include predicting the need for permanent pacemaker implantation [117,118], anticipating high-impact postoperative morbidities such as acute kidney injury to enable targeted intervention [119], and forecasting long-term outcomes like structural valve deterioration requiring reintervention [120,121]. Additionally, we see this benchmark as a foundation that can be extended beyond discrimination to also evaluate uncertainty estimation performance to assess predictive trustworthiness.

## Figures and Tables

**Table 1:** Selected characteristics of VR surgery dataset *VR-general*.

Characteristic	Value
**Patient Demographics**	
Number of Patients	3890
Number of Hospital Admissions	12,205
Age at time of VR (years), 5th/50th/95th percentile	45/72/89
Gender	Male: 2378 (61%)
	Female: 1512 (39%)
**Risk Factors**	
Smoker	1042 (27%)
Diabetes Mellitus	797 (20%)
**Comorbidities**	
Anemia	1090 (28%)
Atrial Fibrillation	1159 (30%)
Chronic Kidney Disease	763 (20%)
Chronic Obstructive Pulmonary Disease	396 (10%)
Renal Failure	795 (20%)
**Valve Replacement Procedure**	
Aortic valve	3224 (83%)
Mitral valve	613 (16%)
Pulmonary valve	50 (1%)
Tricuspid valve	3 (<1%)
**Hospitalization Statistics**	
Number of Hospital Admissions per patient, 5th/50th/95th percentile	1/2/10
Length of Hospital Stay (days), 5th/50th/95th percentile	0.8/5.0/20.5
Single ICU admission during VR Hospital Stay	3443 (89%)
Two or more ICU admissions during VR Hospital Stay	447 (11%)
In-hospital Mortality	32 (0.8%)
**Common Postoperative Complications**	
Stroke	424 (11%)
Mechanical Complications	29 (1%)
Thromboembolic Events	418 (11%)
Infection	132 (3%)
Endocarditis	250 (6%)

**Table 2:** ICU readmission prediction performance on the *VR-pred* dataset for a Transformer trained on time-stamped token sequences (SeqT), a Transformer trained on summary statistics (NonSeqT), and XGBoost also trained on summary statistics. Mean and standard deviation were obtained using bootstrapping (1000 populations of dataset size) from the pooled predictions of a stratified 10-fold cross-validation.

Model	AUROC		AUPRC	
	μ	σ	μ	σ
SeqT	0.869	0.013	0.686	0.024
NonSeqT	0.821	0.015	0.560	0.021
XGBoost	0.825	0.016	0.625	0.030

**Table 3:** Corrected resampled *t*-test (Nadeau–Bengio) on split-wise paired deltas across RK=50 splits. Reported are the mean difference in scores $\bar{d}$, the corrected 95% CI, and corrected one-sided *p*-value. Positive deltas indicate the first model outperforms the second.

Comparison	AUROC			AUPRC		
	$\bar{d}$	**95% CI (corr.)**	p	$\bar{d}$	**95% CI (corr.)**	p
SeqT − NonSeqT	0.03	[0.00,0.06]	0.045	0.11	[0.04,0.17]	0.001
SeqT − XGB	0.03	[−0.01,0.07]	0.061	0.04	[−0.04,0.12]	0.157
XGB − NonSeqT	0.00	[−0.03,0.03]	0.622	0.07	[0.02,0.11]	0.005

**Table 4:** Category-level feature relevance. Category-level aggregated feature importances using $\phi_{k}$ correlation coefficient, SHAP values from the XGBoost model and *Integrated Gradients* (IG) scores from the sequential Transformer model. For IG and SHAP, all scores were averaged across 10-fold cross-validation. Shown are the relative mean feature importance per category, ${\bar{I}}_{f}$, the relative cumulated category importance, $I_{c}$, and the size-normalized category importance, $I_{c}^{★}$.

Category	$\phi_{k}$			SHAP			IG		
	${\bar{I}}_{f}$	$I_{c}$	$I_{c}^{★}$	${\bar{I}}_{f}$	$I_{c}$	$I_{c}^{★}$	${\bar{I}}_{f}$	$I_{c}$	$I_{c}^{★}$
Procedures	0.17	0.38	1.44	0.03	0.12	0.45	0.04	0.24	0.91
Historic Diagn.	0.10	0.27	0.44	0.01	0.01	0.01	0.01	0.09	0.14
Lab	0.31	0.19	17.19	0.69	0.74	64.60	0.28	0.50	43.55
Medication	0.17	0.14	1.34	0.10	0.13	1.25	0.06	0.12	1.15
Phys. Monitoring	0.08	0.02	3.50	0.00	0.00	0.12	0.09	0.04	8.65
Pat. Demographics	0.17	0.00	1.01	0.17	0.01	12.25	0.52	0.01	19.32

**Figure A1:**
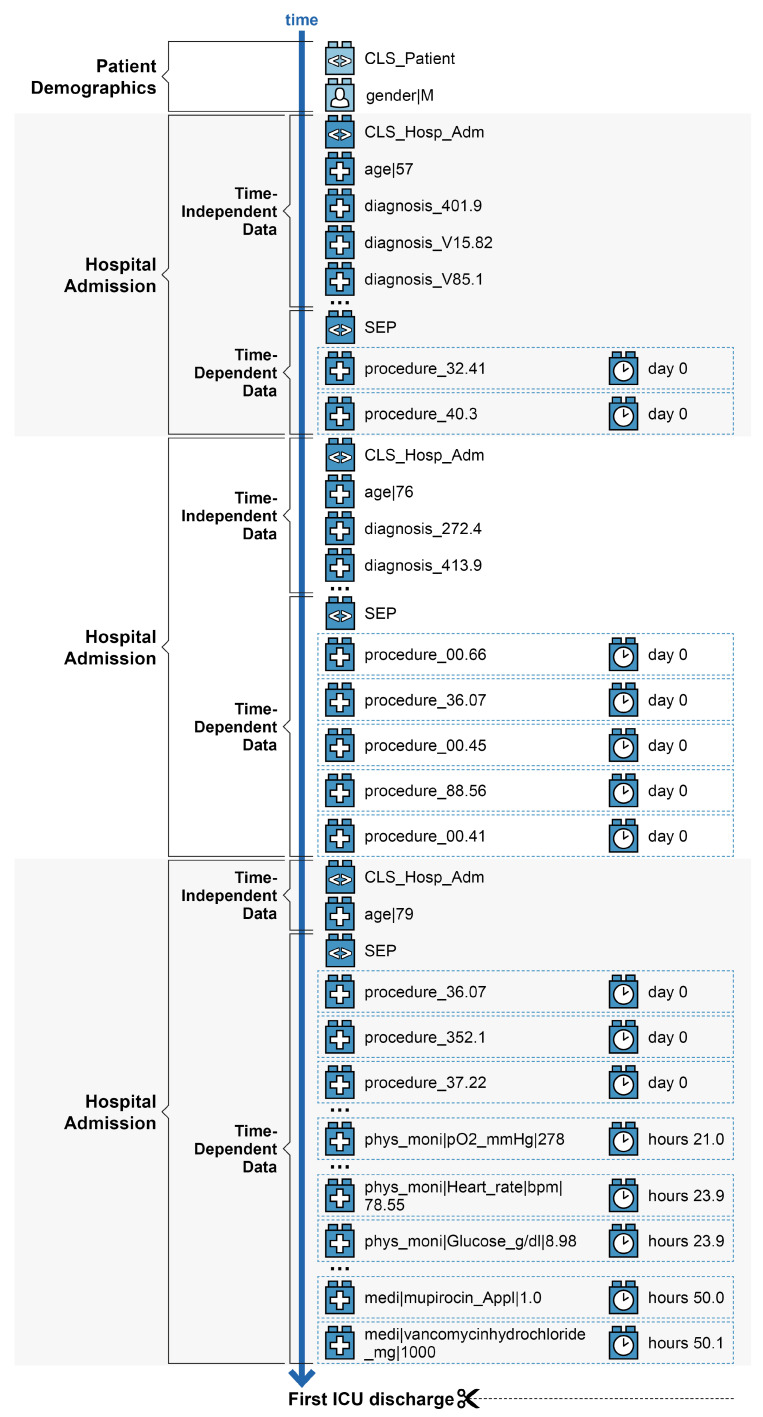
Representation of a patient’s EHR data as a token sequence. Features were altered due to privacy concerns, maintaining a similar clinical pathway.

**Figure A2:**
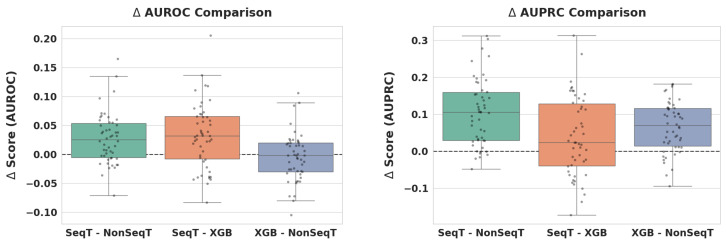
Boxplot of split-wise deltas for AUROC and AUPRC. Colored boxes represent the interquartile range (IQR), spanning from the 25th to the 75th percentile. Whiskers extend to 1.5 times the IQR beyond the box boundaries.

## Data Availability

Data were derived from the MIMIC-IV database (v3.1), requiring credentialed public access via PhysioNet (https://physionet.org/content/mimiciv/, accessed on 20 December 2024). To comply with the MIMIC-IV Data Use Agreement, patient-level data are not shared directly. The specific datasets, *VR-general* and *VR-pred*, were generated using our custom processing pipeline; code is available at https://git.iccas.de/valve-replacement-risk-stratification/dataset-pipeline-vr, accessed on 29 January 2026. Researchers with their own approved access to MIMIC-IV can utilize this codebase to regenerate the cohorts locally, ensuring full reproducibility without violating data sharing restrictions.

## Appendix A. Valve Replacement Procedures

In Table A1 we present an extensive list of ICD-PCS codes used to identify patients who have undergone VR procedures in the MIMIC-IV database. These patients were integrated into our cohort. Since MIMIC-IV contains ICD-9 and ICD-10 codes, both formats were included. Reconstructive heart valve operations (i.e., native valve repair without prosthetic replacement) were excluded.

**Table A1:** ICD-9 and ICD-10-PCS codes for heart VR procedures used for cohort definition. Asterisks (*) indicate codes applicable to all heart valves. To specify the valve, replace the asterisk (*) with one of the following: F (Aortic), G (Mitral), H (Pulmonary), or J (Tricuspid).

ICD Code	Description
3505	Endovascular replacement of aortic valve
3506	Transapical replacement of aortic valve
3521	Open and other replacement of aortic valve with tissue graft
3522	Open and other replacement of aortic valve
3523	Open and other replacement of mitral valve with tissue graft
3524	Open and other replacement of mitral valve
3507	Endovascular replacement of pulmonary valve
3508	Transapical replacement of pulmonary valve
3525	Open and other replacement of pulmonary valve with tissue graft
3526	Open and other replacement of pulmonary valve
3527	Open and other replacement of tricuspid valve with tissue graft
3528	Open and other replacement of tricuspid valve
	**Replacement of Valve with: **
02RF08N	Zooplastic Tissue, using Rapid Deployment Technique, Open Approach
02RF38N	Zooplastic Tissue, using Rapid Deployment Technique, Percutaneous Approach
02RF48N	Zooplastic Tissue, using Rapid Deployment Technique,
	Percutaneous Endoscopic Approach
X2RF032	Zooplastic Tissue, Rapid Deployment Technique, Open Approach
X2RF332	Zooplastic Tissue, Rapid Deployment Technique, Percutaneous Approach
X2RF432	Zooplastic Tissue, Rapid Deployment Technique, Percutaneous Endoscopic Approach
02RH38L	Zooplastic Tissue, In Existing Conduit, Percutaneous Approach
02RH38M	Zooplastic Tissue, Native Site, Percutaneous Approach
02R*07Z	Autologous Tissue Substitute, Open Approach
02R*08Z	Zooplastic Tissue, Open Approach
02R*0JZ	Synthetic Substitute, Open Approach
02R*0KZ	Nonautologous Tissue Substitute, Open Approach
02R*37H	Autologous Tissue Substitute, Transapical, Percutaneous Approach
02R*37Z	Autologous Tissue Substitute, Percutaneous Approach
02R*38H	Zooplastic Tissue, Transapical, Percutaneous Approach
02R*38Z	Zooplastic Tissue, Percutaneous Approach
02R*3JH	Synthetic Substitute, Transapical, Percutaneous Approach
02R*3JZ	Synthetic Substitute, Percutaneous Approach
02R*3KH	Nonautologous Tissue Substitute, Transapical, Percutaneous Approach
02R*3KZ	Nonautologous Tissue Substitute, Percutaneous Approach
02R*47Z	Autologous Tissue Substitute, Percutaneous Endoscopic Approach
02R*48Z	Zooplastic Tissue, Percutaneous Endoscopic Approach
02R*4JZ	Synthetic Substitute, Percutaneous Endoscopic Approach
02R*4KZ	Nonautologous Tissue Substitute, Percutaneous Endoscopic Approach

## Appendix B. VR-General Dataset

Table A2 summarizes clinically relevant features for VR surgery, defined through a physician-led selection process guided by established guidelines and refined iteratively. The *VR-general* dataset was constructed by mapping corresponding variables from MIMIC-IV to these high-level clinical feature definitions. Further details on the feature selection process and its rationale are provided in Section 3.1.

**Table A2:** High-level medical features relevant to VR surgery.

Category	Details
**General Information**	
Gender	
Age	
Height	
Weight	
BMI	
History of Tobacco Use	Smoking Status, Quit Date if Applicable
**Patient History**	
Diagnoses	All Diagnoses Recorded During Any Hospitalization
Procedures	All Procedures Recorded During Any Hospitalization
**Parameters During VR Hospitalization**	
*Laboratory Measurements*	
Kidney Retention Parameters	Creatinine, Urea, eGFR
Electrolytes	Potassium, Sodium, Magnesium, Phosphate, Calcium, Chloride, Osmolality
Inflammatory Markers	C-Reactive Protein (CRP)
Liver Function Tests	ALT, AST, Albumin, Bilirubin, Alkaline~Phosphatase
Hematology	Hemoglobin, Hematocrit, MCHC, RDW, WBC, Lymphocytes, Basophils,
	Eosinophils, Neutrophils, Monocytes
Cardiac Markers	BNP, Troponin I, Troponin T, CK-MB, LDH
Diabetes Markers	Blood Glucose, HbA1c
Coagulation Parameters	INR, Thrombin Time, PT, aPTT, Fibrinogen, Platelets
Lipid Profile	LDL, HDL, Triglycerides, Total Cholesterol
Blood Gas Analysis	Anion Gap, Bicarbonate, Base Excess, Oxygen Saturation, $pO_{2}$,
	$pCO_{2}$, pH, Lactate
*Medication*	All Medications
*Procedures*	All Procedures
*Physiological Monitoring Data*	Postoperative
Lung Function	Respiratory Rate, Vital Capacity, Resistance, Arterial $O_{2}$ pressure,
	Arterial $O_{2}$ Saturation, Arterial $CO_{2}$ Pressure
Heart Function	Left Atrial Pressure, Cardiac Output, EF
*Vital Signs*	
Blood Pressure	Non Invasive (on Ward)/Central Venous Pressure,
	Pulmonary Artery Pressure, Arterial Blood Pressure (in ICU)
Heart Rate	
Temperature	

## Appendix C. Tokenization Scheme

We describe the scheme used for tokenization of a patient’s EHR data. First, we incorporate patient demographics, such as gender or height. Next, we order each patient’s hospital visits chronologically. To mark the beginning of a new hospital admission, we introduce a special control token, *CLS_Hosp_Adm*, followed by time-independent features, such as diagnostic codes registered during the stay. Another control token, *SEP*, introduces a time-ordered sequence of time-dependent features. Binary medical events, such as the occurrence of a procedure, are represented by a single token. This pattern is repeated for subsequent hospital admissions to tokenize all patient data. Due to the daily resolution of procedure codes, these tokens are placed at the beginning of their respective daily sequences by convention. This placement does not reflect the true intra-day chronological order of events and serves only as pragmatic workaround. Continuous outcome variables such as laboratory measurements or dose-specific medications are discretized into value-specific tokens using quantile-based binning. This approach was selected over alternative strategies, most notably continuous value integration via learned embeddings or uniform-range binning. Below, we outline the advantages and limitations of this design choice. Continuous modeling, which maps scalar values into the Transformer’s embedding space via learnable projections, offers the advantage of preserving numerical precision and reducing vocabulary size (as it separates the medical concept from its numerical value). However, our discretization strategy aligns well with clinical practice, where decisions are often based on discrete thresholds (e.g., guidelines for hypertension) or standardized dosage units rather than actual continuous values. By treating these ranges as distinct tokens, patient data representation remains directly connected to the patient’s relative deviation from the population norm, making it a plausible representation for patient stratification tasks. Furthermore, the binning approach transforms heterogeneous patient data into uniform, sentence-like discrete token sequences, allowing the application of standard Transformer architectures without the need for custom engineering to handle hybrid data types. We specifically employed quantile-based binning to distribute values such that each bin contains an approximately equal number of samples. This ensures that all tokens are sufficiently represented within the training data. This is particularly important for capturing clinically critical outliers (e.g., extreme hypotension) that might be underrepresented in uniform binning schemes. Additionally, quantile-based binning aggregates extreme outliers (e.g., caused by decimal errors during documentation) into a single terminal bin. This prevents the creation of numerous sparsely populated intermediate bins, resulting in a balanced input distribution that enhances training stability.

Figure A1 illustrates an exemplary token sequence, where numerical identifiers represent ICD codes. In the following, we describe this sequence, particularly outlining relations to the patient’s VR. During the patient’s first hospital admission at age 57 for a malignant lung tumor, an aortic valve disease diagnosis was made. Although no direct causal link exists between valvular heart disease and lung cancer, their co-occurrence is plausible due to shared epidemiological risk factors such as smoking, hypertension, and obesity, which predispose individuals to both cardiovascular and oncological diseases [122]. The second hospital admission, at age 76, indicated significant cardiovascular deterioration: in addition to hyperlipidemia, the patient developed angina pectoris, requiring stent placement. At 79, during the third hospitalization, the patient underwent an aortic valve replacement (AVR). The urgency of the patient’s clinical status is indicated by the fact that cardiac catheterization was performed less than 24 h before AVR. For elective cases, this diagnostic procedure typically occurs several weeks or months before surgery [123]. Postoperative clinical monitoring included parameters such as oxygen, glucose, and heart rate. Notably, the patient was identified as a carrier of Methicillin-resistant Staphylococcus aureus (MRSA) and underwent preoperative decolonization with intranasal mupirocin. Consequently, the standard prophylactic regimen was supplemented with targeted perioperative vancomycin prophylaxis, in accordance with established guidelines for MRSA-colonized surgical patients [104].

## Appendix D. Postoperative Complications

In Table A2 we present the ICD-9-CM and ICD-10-CM codes used to identify common postoperative complications following VR procedures (see Figure 3A).

**Table A3:** ICD-9-CM and ICD-10-CM codes of postoperative complications associated with valve replacement procedures. Asterisks (*) denote the inclusion of all valid suffixes. Numbers in curly braces (e.g., {1,3,4}) indicate that any of the enclosed digits can occupy that position in the code.

ICD Code	Description
**Stroke ** (Only ischemic strokes are considered here, as valve prosthesis and potential postoperative atrial fibrillation increase the risk of embolisms. Hemorrhagic strokes, despite an increased risk, are excluded as they primarily result from anticoagulation therapy, not directly from the valve material or surgery.)	
433. *	Occlusion and stenosis of precerebral arteries
434. *	Occlusion of cerebral arteries
435. *	Transient cerebral ischemia
G45. *	Transient cerebral ischemic attacks and related syndromes
I63. *	Cerebral infarction
**Thromboembolic Events (other than Stroke)**	
444. *	Arterial embolism and thrombosis
451. *	Phlebitis and thrombophlebitis
452	Portal vein thrombosis
453. *	Other venous embolism and thrombosis
I74. *	Arterial embolism and thrombosis
I80. *	Thrombosis, phlebitis and thrombophlebitis
I81	Portal vein thrombosis
I82. *	Other venous embolism and thrombosis
T82.81 *	Embolism due to cardiac prosthetic devices, implants and grafts
T82.86 *	Thrombosis due to cardiac prosthetic devices, implants and grafts
**Infection**	
996.61	Infection and inflammatory reaction due to cardiac device, implant, and graft
T82.6 *	Infection and inflammatory reaction due to cardiac valve prosthesis
**Endocarditis**	
421. *	Acute and subacute endocarditis
424.9 *	Endocarditis valve unspecified
I33. *	Acute and subacute endocarditis
**Mechanical Complication**	
996.02	Mechanical complication due to heart valve prosthesis
T82.0 *	Mechanical complication of heart valve prosthesis
**Other Complications**	
996.71	Other complications due to heart valve prosthesis
T82.8{2,3,4,5,9} *	Other complications of cardiac and vascular prosthetic devices, implants and grafts
T82.9 *	Unspecified complication of cardiac and vascular prosthetic device, implant and graft

## Appendix E. Detailed Feature Relevance and Attribution

To complement the category-level feature relevance analysis presented in the main manuscript, we provide the top-three features per category as ranked by the three different feature importance assessment methods in Table A4, Table A5 and Table A6. For a discussion of the clinical plausibility of these features, we refer to Section 5.

**Table A4:** Feature relevance: $\phi_{k}$ correlation. Top three features per category, ranked by $\phi_{k}$ correlation with ICU readmission. The mean score for all features in the category is also shown. All reported correlations were statistically significant (p<0.05) and *p*-values are omitted for clarity. Features in bold are also top-ranked by IG and SHAP.

Category	Feature	Feature Importance
Procedure	Introduction of Nutritional Substance	
	to Upper GI (3E0G76Z)	0.33
	Continuous Respiratory Mechanical	
	Ventilation > 96 h (5A1955Z)	0.27
	Endotracheal Airway Insertion (0BH17EZ)	0.27
Historical Diagnoses	Severe Sepsis with Septic Shock (R65.21)	0.13
	Ischemic Cardiomyopathy (I25.5)	0.12
	Unspecified Viral Hepatitis C	
	without Hepatic Coma (070.70)	0.10
Lab	Base Excess Blood Gas	0.39
	pH Blood Gas	0.33
	$pO_{2}$ Blood Gas	0.33
Medication	Docusate Sodium	0.44
	**Cefazolin Sodium**	**0.32**
	Propofol	0.25
Physiological Monitoring Data	Pulmonary Artery Pressure diastolic	0.07
	**Arterial Blood Pressure systolic**	**0.05**
	Pulmonary Artery Pressure mean	0.05
Patient Demographics	**Age at Hospital Admission**	**0.08**
	**Gender**	**0.06**

**Table A5:** Feature relevance: integrated gradients. Top three features per category for the time-series Transformer model, ranked by IG scores. All scores were averaged during 10-fold cross-validation. The mean score for all features in the category is also shown. Features in bold are also top-ranked by $\phi_{k}$ and SHAP.

Category	Feature	Feature Importance
Procedure	Pericardiocentesis (0W9D3ZZ)	37.0
	Fluoroscopy of Mammary	
	Bypass Graft (B2181ZZ)	0.45
	Injection or infusion of therapeutic	
	or prophylactic substance (99.29)	0.37
Historical Diagnoses	Anticoagulants causing	
	adverse effects (E934.2)	0.05
	Personal history of other	
	malignant neoplasm (Z85.038)	0.05
	Cardiogenic shock (R57.0)	0.05
Lab	Chloride Blood Gas	0.90
	Sodium Blood Gas	0.82
	Hematocrit Blood Gas	
Medication	**Cefazolin Sodium**	**0.47 **
	Metoprolol Tartrate	0.34
	Insulin Glargine	0.30
Physiological Monitoring Data	**Arterial Blood Pressure systolic**	**0.36**
	Arterial Pressure mean	0.35
	Cardiac Output (CCO)	0.28
Patient Demographics	**Age at Hospital Admission**	**0.98**
	**Gender**	**0.95**

**Table A6:** Feature relevance: SHAP. Top three features per category for the XGBoost model, ranked by SHAP values. All scores were averaged during 10-fold cross-validation. The mean score for all features in the category is also shown. Features in bold are also top-ranked by $\phi_{k}$ and IG.

Category	Feature	Feature Importance
Procedure	Introduction of Nutritional	
	Substance to Upper GI (3E0G76Z)	0.32
	Insertion of Infusion Device into	
	Superior Vena Cava (02HV33Z)	0.26
	Continuous Respiratory Mechanical	
	Ventilation > 96 h (5A1955Z)	0.16
Historical Diagnoses	Aortic valve disorders (424.1)	0.02
	Hypertensive chronic kidney disease (I12.9)	0.01
	Asthma, unspecified type (493.90)	0.01
Lab	pH Blood Gas	1.00
	$pO_{2}$ Blood Gas	0.98
	Glucose Blood Gas	0.85
Medication	Docusate Sodium	0.68
	**Cefazolin Sodium**	**0.67**
	Metoprolol Tartrate	0.25
Physiological Monitoring Data	Heart Rate	0.00
	**Arterial Blood Pressure systolic**	**0.00**
	Heart rate Alarm - High	0.00
Patient Demographics	**Age at Hospital Admission**	0.24
	**Gender**	**0.22**

## Appendix F. Statistical Tests for the Benefit of Temporal EHR Sequences

This appendix reports statistical tests comparing the sequential Transformer (SeqT), the non-sequential Transformer trained on aggregated features (NonSeqT), and the XGBoost baseline (XGB). All models were evaluated under identical R=5 repetitions of stratified K=10-fold patient-level cross-validation (RK=50 test splits).

For each split (r,k) and metric M∈{AUROC,AUPRC}, let $M_{r,k}\left(m\right)$ denote the test-fold score of model m∈{SeqT,NonSeqT,XGB}. We form paired deltas on the *same* test fold:

(A1) $$\begin{array}{cc} d_{\mathrm{temp},r,k}^{\left(M\right)} & =M_{r,k}\left(\mathrm{SeqT}\right)-M_{r,k}\left(\mathrm{NonSeqT}\right), \end{array}$$

(A2) $$\begin{array}{cc} d_{\mathrm{base},r,k}^{\left(M\right)} & =M_{r,k}\left(\mathrm{SeqT}\right)-M_{r,k}\left(\mathrm{XGB}\right), \end{array}$$

(A3) $$\begin{array}{cc} d_{\mathrm{arch},r,k}^{\left(M\right)} & =M_{r,k}\left(\mathrm{XGB}\right)-M_{r,k}\left(\mathrm{NonSeqT}\right). \end{array}$$

Because repeated *K*-fold CV yields correlated split-wise estimates, we apply the corrected resampled *t*-test [64] to each set of deltas ${\left\{d_{\cdot ,r,k}^{\left(M\right)}\right\}}_{r,k}$. For a given comparison and metric *M* we compute

$${\bar{d}}^{\left(M\right)}=\frac{1}{RK}\sum_{r=1}^{R}\sum_{k=1}^{K}d_{r,k}^{\left(M\right)},$$

and the sample variance $s_{d}^{2}$ of the RK deltas. The corrected standard error is

$${\mathrm{SE}}_{\mathrm{corr}}=\sqrt{\left(\frac{1}{RK}+\frac{1}{K-1}\right)s_{d}^{2}},$$

yielding the corrected t-statistic $t_{\mathrm{corr}}={\bar{d}}^{\left(M\right)}/{\mathrm{SE}}_{\mathrm{corr}}$. We report corrected *p*-values and corrected 95% confidence intervals for ${\bar{d}}^{\left(M\right)}$.

We use a one-sided directional test consistent with the stated hypothesis that temporal information improves performance:

$$H_{0}:E\left[d^{\left(M\right)}\right]\leq 0\;\mathrm{vs}.\;H_{1}:E\left[d^{\left(M\right)}\right]>0.$$

Figure A2 shows the boxplots of the split-wise mean deltas.

## References

[1] Misawa Y. (2015). Valve-related complications after mechanical heart valve implantation. Surg. Today.

[2] Del Val D., Abdel-Wahab M., Mangner N., Durand E., Ihlemann N., Urena M., Pellegrini C., Giannini F., Gasior T., Wojakowski W. (2021). Stroke complicating infective endocarditis after transcatheter aortic valve replacement. J. Am. Coll. Cardiol..

[3] Dodge A., Hurni M., Ruchat P., Stumpe F., Fischer A., Van Melle G., Sadeghi H. (1995). Surgery in native valve endocarditis: Indications, results and risk factors. Eur. J. Cardio-Thorac. Surg..

[4] Obafemi T., Mullis D., Bajaj S., Krishna P., Boyd J. (2023). Results following implementation of a cardiac surgery ERAS protocol. PLoS ONE.

[5] Ljungqvist O., Scott M., Fearon K.C. (2017). Enhanced recovery after surgery: A review. JAMA Surg..

[6] Jensen P.B., Jensen L.J., Brunak S. (2012). Mining Electronic Health Records: Towards Better Research Applications and Clinical Care. Nat. Rev. Genet..

[7] Howe J.L., Adams K.T., Hettinger A.Z., Ratwani R.M. (2018). Electronic Health Record Usability Issues and Potential Contribution to Patient Harm. J. Am. Med. Assoc..

[8] Goodfellow I., Bengio Y., Courville A. (2016). Deep Learning.

[9] Xiao C., Choi E., Sun J. (2018). Opportunities and Challenges in Developing Deep Learning Models Using Electronic Health Records Data: A Systematic Review. J. Am. Med. Inform. Assoc..

[10] Roques F., Michel P., Goldstone A., Nashef S. (2003). The logistic euroscore. Eur. Heart J..

[11] Nashef S.A., Roques F., Sharples L.D., Nilsson J., Smith C., Goldstone A.R., Lockowandt U. (2012). Euroscore ii. Eur. J. Cardio-Thorac. Surg..

[12] Dhillon G., Hassan H., Andra C.A., Zainal S., Raynaldo A.H., Haykal T.B. (2022). STS (Society of Thoracic Surgeon) Score as a Predictor for Major Adverse Cardiovascular Events in Patients Undergone Coronary Artery Bypass Surgery During Admission in Haji Adam Malik General Hospital Medan. Zenodo.

[13] Kilic A., Goyal A., Miller J.K., Gleason T.G., Dubrawksi A. (2021). Performance of a machine learning algorithm in predicting outcomes of aortic valve replacement. Ann. Thorac. Surg..

[14] Jiang H., Liu L., Wang Y., Ji H., Ma X., Wu J., Huang Y., Wang X., Gui R., Zhao Q. (2021). Machine learning for the prediction of complications in patients after mitral valve surgery. Front. Cardiovasc. Med..

[15] Agasthi P., Ashraf H., Pujari S.H., Girardo M.E., Tseng A., Mookadam F., Venepally N.R., Buras M., Khetarpal B.K., Allam M. (2021). Artificial intelligence trumps TAVI2-SCORE and CoreValve score in predicting 1-year mortality post-transcatheter aortic valve replacement. Cardiovasc. Revasc. Med..

[16] Hoffmann J., Mas-Peiro S., Berkowitsch A., Boeckling F., Rasper T., Pieszko K., De Rosa R., Hiczkiewicz J., Burchardt P., Fichtlscherer S. (2020). Inflammatory signatures are associated with increased mortality after transfemoral transcatheter aortic valve implantation. ESC Heart Fail..

[17] Kwiecinski J., Dabrowski M., Nombela-Franco L., Grodecki K., Pieszko K., Chmielak Z., Pylko A., Hennessey B., Kalinczuk L., Tirado-Conte G. (2023). Machine learning for prediction of all-cause mortality after transcatheter aortic valve implantation. Eur. Heart J. Qual. Care Clin. Outcomes.

[18] Lertsanguansinchai P., Chokesuwattanaskul R., Petchlorlian A., Suttirut P., Buddhari W., Chula TAVI Team (2023). Machine learning-based predictive risk models for 30-day and 1-year mortality in severe aortic stenosis patients undergoing transcatheter aortic valve implantation. Int. J. Cardiol..

[19] Al-Farra H., Ravelli A.C., Henriques J.P., Houterman S., de Mol B.A., Abu-Hanna A., Committee N.T.R. (2022). Development and validation of a prediction model for early mortality after transcatheter aortic valve implantation (TAVI) based on the Netherlands Heart Registration (NHR): The TAVI-NHR risk model. Catheter. Cardiovasc. Interv..

[20] Leha A., Huber C., Friede T., Bauer T., Beckmann A., Bekeredjian R., Bleiziffer S., Herrmann E., Möllmann H., Walther T. (2023). Development and validation of explainable machine learning models for risk of mortality in transcatheter aortic valve implantation: TAVI risk machine scores. Eur. Heart J. Digit. Health.

[21] Orfanoudaki A., Giannoutsou A., Hashim S., Bertsimas D., Hagberg R.C. (2022). Machine learning models for mitral valve replacement: A comparative analysis with the Society of Thoracic Surgeons risk score. J. Card. Surg..

[22] Edwards F.H., Peterson E.D., Coombs L.P., DeLong E.R., Jamieson W.E., Shroyer A.L.W., Grover F.L. (2001). Prediction of operative mortality after valve replacement surgery. J. Am. Coll. Cardiol..

[23] Hernandez-Suarez D.F., Kim Y., Villablanca P., Gupta T., Wiley J., Nieves-Rodriguez B.G., Rodriguez-Maldonado J., Feliu Maldonado R., da Luz Sant’Ana I., Sanina C. (2019). Machine learning prediction models for in-hospital mortality after transcatheter aortic valve replacement. Cardiovasc. Interv..

[24] Wang J., Zhu J., Li H., Wu S., Li S., Yao Z., Zhu T., Tang B., Tang S., Liu J. (2025). Multimodal Visualization and Explainable Machine Learning–Driven Markers Enable Early Identification and Prognosis Prediction for Symptomatic Aortic Stenosis and Heart Failure With Preserved Ejection Fraction After Transcatheter Aortic Valve Replacement: Multicenter Cohort Study. J. Med Internet Res..

[25] Johnson A., Bulgarelli L., Pollard T., Horng S., Celi L.A., Mark R. Mimic-iv. PhysioNet. 2020. pp. 49–55. https://physionet.org/content/mimiciv/1.0/.

[26] Johnson A.E., Bulgarelli L., Shen L., Gayles A., Shammout A., Horng S., Pollard T.J., Hao S., Moody B., Gow B. (2023). MIMIC-IV, a freely accessible electronic health record dataset. Sci. Data.

[27] von Ballmoos M.C.W., Kaneko T., Iribarne A., Kim K.M., Arghami A., Fiedler A., Habib R., Parsons N., Elhalabi Z., Krohn C. (2024). The society of thoracic surgeons adult cardiac surgery database: 2023 update on procedure data and research. Ann. Thorac. Surg..

[28] Taha A., David A., Ragnarsson S., Szamlewski P., Jamaly S., Smith J.G., Nielsen S.J., Jeppsson A., Martinsson A. (2025). Pacemaker implantation after cardiac surgery: A contemporary, nationwide perspective. Heart.

[29] Jung J.C., Jang M.J., Hwang H.Y. (2019). Meta-analysis comparing mitral valve repair versus replacement for degenerative mitral regurgitation across all ages. Am. J. Cardiol..

[30] Frank L., Mach F., Yvo M., Carballo D., Konstantinos C., Bäck M., Benetos A., Biffi A., Boavida J., Capodanno D. (2021). ESC Guidelines on cardiovascular disease prevention in clinical practice: Developed by the Task Force for cardiovascular disease prevention in clinical practice with representatives of the European Society of Cardiology and 12 medical societies With the special contribution of the European Association of Preventive Cardiology (EAPC). Eur. Heart J..

[31] World Health Organization (2011). Global atlas on cardiovascular disease prevention and control. Global Atlas on Cardiovascular Disease Prevention and Control.

[32] Piñón M., Paredes E., Acuña B., Raposeiras S., Casquero E., Ferrero A., Torres I., Legarra J.J., Pradas G., Barreiro-Morandeira F. (2019). Frailty, disability and comorbidity: Different domains lead to different effects after surgical aortic valve replacement in elderly patients. Interact. Cardiovasc. Thorac. Surg..

[33] Lee K.S., Park D.I., Lee J., Oh O., Kim N., Nam G. (2023). Relationship between comorbidity and health outcomes in patients with heart failure: A systematic review and meta-analysis. BMC Cardiovasc. Disord..

[34] Clough R.A., Leavitt B.J., Morton J.R., Plume S.K., Hernandez F., Nugent W., Lahey S.J., Ross C.S., O’Connor G.T., Group N.N.E.C.D.S. (2002). The effect of comorbid illness on mortality outcomes in cardiac surgery. Arch. Surg..

[35] Cornelissen C.G., Frechen D.A., Schreiner K., Marx N., Krüger S. (2013). Inflammatory parameters and prediction of prognosis in infective endocarditis. BMC Infect. Dis..

[36] Wilczek K., Bujak K., Reguła R., Chodór P., Osadnik T. (2015). CARDIAC SURGERY Risk factors for paravalvular leak after transcatheter aortic valve implantation. Kardiochirurgia I Torakochirurgia Pol. J. Thorac. Cardiovasc. Surg..

[37] Côté N., Pibarot P., Clavel M.A. (2017). Incidence, risk factors, clinical impact, and management of bioprosthesis structural valve degeneration. Curr. Opin. Cardiol..

[38] Brosin J. (1995). Untersuchung zur Häufigkeit Postoperativer Blutungen nach Dermatochirurgischen Eingriffen von Patienten Unter Antithrombotischer Medikation: Eine Retrospektive Analyse. Ph.D. Thesis.

[39] O’Rourke D.J., Palac R.T., Malenka D.J., Marrin C.A., Arbuckle B.E., Plehn J.F. (2001). Outcome of mild periprosthetic regurgitation detected by intraoperative transesophageal echocardiography. J. Am. Coll. Cardiol..

[40] Matteucci M., Ferrarese S., Cantore C., Massimi G., Facetti S., Mantovani V., Cappabianca G., Fina D., Lorusso R., Beghi C. (2020). Early aortic paravalvular leak after conventional cardiac valve surgery: A single-center experience. Ann. Thorac. Surg..

[41] Lonn E. (2001). The use of surrogate endpoints in clinical trials: Focus on clinical trials in cardiovascular diseases. Pharmacoepidemiol. Drug Saf..

[42] Christensen R., Ciani O., Manyara A.M., Taylor R.S. (2024). Surrogate endpoints: A key concept in clinical epidemiology. J. Clin. Epidemiol..

[43] Litmathe J., Kurt M., Feindt P., Gams E., Boeken U. (2009). Predictors and outcome of ICU readmission after cardiac surgery. Thorac. Cardiovasc. Surg..

[44] Vaswani A., Shazeer N., Parmar N., Uszkoreit J., Jones L., Gomez A.N., Kaiser Ł., Polosukhin I. (2017). Attention is all you need. Adv. Neural Inf. Process. Syst..

[45] Li Y., Rao S., Solares J.R.A., Hassaine A., Ramakrishnan R., Canoy D., Zhu Y., Rahimi K., Salimi-Khorshidi G. (2020). BEHRT: Transformer for electronic health records. Sci. Rep..

[46] Lindenmeyer A., Blattmann M., Franke S., Neumuth T., Schneider D. (2025). Towards Trustworthy AI in Healthcare: Epistemic Uncertainty Estimation for Clinical Decision Support. J. Pers. Med..

[47] Liu P.J., Saleh M., Pot E., Goodrich B., Sepassi R., Kaiser L., Shazeer N. (2018). Generating wikipedia by summarizing long sequences. arXiv.

[48] Pang C., Jiang X., Kalluri K.S., Spotnitz M., Chen R., Perotte A., Natarajan K. CEHR-BERT: Incorporating temporal information from structured EHR data to improve prediction tasks. Proceedings of the Machine Learning for Health Symposium, PMLR.

[49] Huang K., Altosaar J., Ranganath R. (2019). Clinicalbert: Modeling clinical notes and predicting hospital readmission. arXiv.

[50] Li Y., Mamouei M., Salimi-Khorshidi G., Rao S., Hassaine A., Canoy D., Lukasiewicz T., Rahimi K. (2022). Hi-BEHRT: Hierarchical transformer-based model for accurate prediction of clinical events using multimodal longitudinal electronic health records. IEEE J. Biomed. Health Inform..

[51] Rasmy L., Xiang Y., Xie Z., Tao C., Zhi D. (2021). Med-BERT: Pretrained contextualized embeddings on large-scale structured electronic health records for disease prediction. NPJ Digit. Med..

[52] Kingma D.P., Ba J. (2017). Adam: A method for stochastic optimization (2014). arXiv.

[53] Chen T., Guestrin C. (2016). XGBoost: A scalable tree boosting system. Proceedings of the 22nd ACM SIGKDD International Conference on Knowledge Discovery and Data Mining.

[54] Nwanosike E.M., Conway B.R., Merchant H.A., Hasan S.S. (2022). Potential applications and performance of machine learning techniques and algorithms in clinical practice: A systematic review. Int. J. Med. Inform..

[55] Hong W., Zhou X., Jin S., Lu Y., Pan J., Lin Q., Yang S., Xu T., Basharat Z., Zippi M. (2022). A comparison of XGBoost, random forest, and nomograph for the prediction of disease severity in patients with COVID-19 pneumonia: Implications of cytokine and immune cell profile. Front. Cell. Infect. Microbiol..

[56] Lv H., Yang X., Wang B., Wang S., Du X., Tan Q., Hao Z., Liu Y., Yan J., Xia Y. (2021). Machine learning–driven models to predict prognostic outcomes in patients hospitalized with heart failure using electronic health records: Retrospective study. J. Med. Internet Res..

[57] Tanaka M., Akiyama Y., Mori K., Hosaka I., Endo K., Ogawa T., Sato T., Suzuki T., Yano T., Ohnishi H. (2025). Machine learning-based analyses of contributing factors for the development of hypertension: A comparative study. Clin. Exp. Hypertens..

[58] Lu S., Chen R., Wei W., Belovsky M., Lu X. Understanding heart failure patients EHR clinical features via SHAP interpretation of tree-based machine learning model predictions. Proceedings of the AMIA Annual Symposium.

[59] Wang Y.G., Chang H.A., Chen M.H., Tzeng N.S., Narumoto J., Liang C.S., Yeh T.C. (2025). Predicting 5-Year Survival and Mortality in Dementia Patients: A Data-Driven Approach Using XGBoost for Enhanced Care and Resource Allocation. Psychiatry Investig..

[60] Neufang S., Li F., Akhrif A., Beyan O.D. (2025). Toward a fair, gender-debiased classifier for the diagnosis of attention deficit/hyperactivity disorder-a Machine-Learning based classification study. BMC Med. Inform. Decis. Mak..

[61] Baak M., Koopman R., Snoek H., Klous S. (2020). A new correlation coefficient between categorical, ordinal and interval variables with Pearson characteristics. Comput. Stat. Data Anal..

[62] Sundararajan M., Taly A., Yan Q. Axiomatic attribution for deep networks. Proceedings of the International Conference on Machine Learning.

[63] Lundberg S.M., Lee S.I. (2017). A unified approach to interpreting model predictions. Adv. Neural Inf. Process. Syst..

[64] Nadeau C., Bengio Y. (2003). Inference for the generalization error. Mach. Learn..

[65] Lee J.J., Srinivasan R., Ong C.S., Alejo D., Schena S., Shpitser I., Sussman M., Whitman G.J., Malinsky D. (2023). Causal determinants of postoperative length of stay in cardiac surgery using causal graphical learning. J. Thorac. Cardiovasc. Surg..

[66] Messé S.R., Acker M.A., Kasner S.E., Fanning M., Giovannetti T., Ratcliffe S.J., Bilello M., Szeto W.Y., Bavaria J.E., Hargrove W.C. (2014). Stroke after aortic valve surgery: Results from a prospective cohort. Circulation.

[67] Alwaqfi N., AlBarakat M.M., Qariouti H., Ibrahim K., Alzoubi N. (2024). Stroke after heart valve surgery: A single center institution report. J. Cardiothorac. Surg..

[68] Waksman R., Minha S. (2014). Stroke after aortic valve replacement: The known and unknown. Circulation.

[69] Panhwar M.S., Ginwalla M., Kalra A., Gupta T., Kolte D., Khera S., Bhatt D.L., Sabik J.F. (2019). Association of acute venous thromboembolism with in-hospital outcomes of coronary artery bypass graft surgery. J. Am. Heart Assoc..

[70] Ho K.M., Bham E., Pavey W. (2015). Incidence of venous thromboembolism and benefits and risks of thromboprophylaxis after cardiac surgery: A systematic review and meta-analysis. J. Am. Heart Assoc..

[71] Laskar N., Bayliss C.D., Kirmani B.H., Chambers J.B., Maier R., Briffa N.P., Cartwright N., Kendall S., Shah B.N., Akowuah E. (2024). Antithrombotic therapy after heart valve surgery: Contemporary practice in the UK. Interdiscip. CardioVascular Thorac. Surg..

[72] Lanz J., Reardon M.J., Pilgrim T., Stortecky S., Deeb G.M., Chetcuti S., Yakubov S.J., Gleason T.G., Huang J., Windecker S. (2021). Incidence and outcomes of infective endocarditis after transcatheter or surgical aortic valve replacement. J. Am. Heart Assoc..

[73] Cahill T.J., Raby J., Jewell P.D., Brennan P.F., Banning A.P., Byrne J., Kharbanda R.K., MacCarthy P.A., Thornhill M.H., Sandoe J.A. (2022). Risk of infective endocarditis after surgical and transcatheter aortic valve replacement. Heart.

[74] Ried I.D., Omran H., Potratz M., Rudolph T.K., Scholtz S., Bleiziffer S., Piper C. (2024). Infective endocarditis after isolated aortic valve replacement: Comparison between catheter-interventional and surgical valve replacement. Clin. Res. Cardiol..

[75] Rubino A.S., Santarpino G., De Praetere H., Kasama K., Dalén M., Sartipy U., Lahtinen J., Heikkinen J., Deste W., Pollari F. (2014). Early and intermediate outcome after aortic valve replacement with a sutureless bioprosthesis: Results of a multicenter study. J. Thorac. Cardiovasc. Surg..

[76] Ali S., Duhan S., Alsaeed T., Atti L., Farooq F., Keisham B., Berry R., Sattar Y., Munir A., Brar V. (2025). The Impact of Periprocedural Prosthetic Valve Leak After Transcatheter Aortic Valve Implantation. Complications.

[77] Foroutan F., Guyatt G.H., O’Brien K., Bain E., Stein M., Bhagra S., Sit D., Kamran R., Chang Y., Devji T. (2016). Prognosis after surgical replacement with a bioprosthetic aortic valve in patients with severe symptomatic aortic stenosis: Systematic review of observational studies. BMJ.

[78] Messori A., Trippoli S., Biancari F. (2013). Early and intermediate survival after transcatheter aortic valve implantation: Systematic review and meta-analysis of 14 studies. BMJ Open.

[79] Nabian M., Badawi O., Amelung P., Atallah L. (2024). 1300: Performance Evaluation of The Discharge Readiness Score Across Eicu Data from 2007 to 2021. Crit. Care Med..

[80] Oakes D.F., Borges I.N.K., Forgiarini L.A., Rieder M.d.M. (2014). Assessment of ICU readmission risk with the Stability and Workload Index for Transfer score. J. Bras. Pneumol..

[81] Wayesa G.A., Berhanu Wedajo M., Demissie W.R., Belay Gizaw A., Hika Gudeta A., Gudina Gula G. (2025). Incidence of prolonged time to tracheal extubation and its associated factors among adult patients undergoing elective surgery at Jimma Medical Center, Jimma, Oromia, Ethiopia, 2024. Perioper. Med..

[82] Camp S., Stamou S., Stiegel R., Reames M., Skipper E., Madjarov J., Velardo B., Geller H., Nussbaum M., Geller R. (2009). Can timing of tracheal extubation predict improved outcomes after cardiac surgery?. HSR Proc. Intensive Care Cardiovasc. Anesth..

[83] Lee H.W., Cho Y.J. (2020). The impact of mechanical ventilation duration on the readmission to intensive care unit: A population-based observational study. Tuberc. Respir. Dis..

[84] Hill A.D., Fowler R.A., Burns K.E., Rose L., Pinto R.L., Scales D.C. (2017). Long-term outcomes and health care utilization after prolonged mechanical ventilation. Ann. Am. Thorac. Soc..

[85] Dadam M.M., Pereira A.B., Cardoso M.R., Carnin T.C., Westphal G.A. (2024). Effect of reintubation within 48 hours on mortality in critically ill patients after planned extubation. Respir. Care.

[86] Welbank T., Kurien M. (2021). To PEG or not to PEG that is the question. Proc. Nutr. Soc..

[87] Kramer A.A., Higgins T.L., Zimmerman J.E. (2012). Intensive care unit readmissions in US hospitals: Patient characteristics, risk factors, and outcomes. Crit. Care Med..

[88] Insights P. (2021). Cardiovascular disease in chronic kidney disease. Circulation.

[89] Lee S.H., Choi K.H., Song Y.B., Jeong D.S., Yang J.H., Kim W.S., Lee Y.T. (2023). Comprehensive assessment of heart failure in patients with preserved ejection fraction undergoing coronary bypass grafting. J. Thorac. Cardiovasc. Surg..

[90] Fu W., Zhao Y., Zhang K., Dai Q., Biekan J., Zheng J., Dong R., Mu J. (2022). Retrospective, observational analysis of cardiac function associated with global preoperative myocardial scar in patients with ischemic cardiomyopathy after coronary artery bypass grafting. J. Thorac. Dis..

[91] Généreux P., Cohen D.J., Pibarot P., Redfors B., Bax J.J., Zhao Y., Prince H., Makkar R.R., Kapadia S., Thourani V.H. (2023). Cardiac damage and quality of life after aortic valve replacement in the PARTNER trials. J. Am. Coll. Cardiol..

[92] Genereux P., Pibarot P., Redfors B., Bax J.J., Zhao Y., Makkar R.R., Kapadia S., Thourani V.H., Mack M.J., Nazif T.M. (2022). Evolution and prognostic impact of cardiac damage after aortic valve replacement. J. Am. Coll. Cardiol..

[93] Shen H.N., Lu C.L., Yang H.H. (2016). Risk of recurrence after surviving severe sepsis: A matched cohort study. Crit. Care Med..

[94] Martin L., Derwall M., Al Zoubi S., Zechendorf E., Reuter D.A., Thiemermann C., Schuerholz T. (2019). The septic heart: Current understanding of molecular mechanisms and clinical implications. Chest.

[95] Care D. (2017). Classification and diagnosis of diabetes. Diabetes Care.

[96] Sarnak M.J., Tighiouart H., Manjunath G., MacLeod B., Griffith J., Salem D., Levey A.S. (2002). Anemia as a risk factor for cardiovascular disease in The Atherosclerosis Risk in Communities (ARIC) study. J. Am. Coll. Cardiol..

[97] Xia H., Shen H., Cha W., Lu Q. (2021). The prognostic significance of anemia in patients with heart failure: A meta-analysis of studies from the last decade. Front. Cardiovasc. Med..

[98] Dal Canto E., Ceriello A., Rydén L., Ferrini M., Hansen T.B., Schnell O., Standl E., Beulens J.W. (2019). Diabetes as a cardiovascular risk factor: An overview of global trends of macro and micro vascular complications. Eur. J. Prev. Cardiol..

[99] Leon B.M., Maddox T.M. (2015). Diabetes and cardiovascular disease: Epidemiology, biological mechanisms, treatment recommendations and future research. World J. Diabetes.

[100] Sarin K., Dhawan N., Shankhyan V.K. (2022). Successful Management of a Case of Sudden Cardiac Arrest in a Postoff-pump Coronary Artery Bypass Graft Surgery Patient on Fourth Postoperative Day and Lessons Learned: A Resuscitation Challenge-A Case Report. Apollo Med..

[101] Modi P., Hassan A., Chitwood W.R. (2008). Minimally invasive mitral valve surgery: A systematic review and meta-analysis. Eur. J. Cardio-Thorac. Surg..

[102] Doenst T., Diab M., Sponholz C., Bauer M., Färber G. (2017). The opportunities and limitations of minimally invasive cardiac surgery. Dtsch. Ärzteblatt Int..

[103] Ilcheva L., Risteski P., Tudorache I., Häussler A., Papadopoulos N., Odavic D., Rodriguez Cetina Biefer H., Dzemali O. (2023). Beyond conventional operations: Embracing the era of contemporary minimally invasive cardiac surgery. J. Clin. Med..

[104] Bratzler D.W., Dellinger E.P., Olsen K.M., Perl T.M., Auwaerter P.G., Bolon M.K., Fish D.N., Napolitano L.M., Sawyer R.G., Slain D. (2013). Clinical practice guidelines for antimicrobial prophylaxis in surgery. Am. J. Health-Syst. Pharm..

[105] Pettersson G.B., Hussain S.T. (2019). Current AATS guidelines on surgical treatment of infective endocarditis. Ann. Cardiothorac. Surg..

[106] Shehabi Y., Bellomo R., Reade M.C., Bailey M., Bass F., Howe B., McArthur C., Seppelt I.M., Webb S., Weisbrodt L. (2012). Early intensive care sedation predicts long-term mortality in ventilated critically ill patients. Am. J. Respir. Crit. Care Med..

[107] Levy D., Laghlam D., Estagnasie P., Brusset A., Squara P., Nguyen L.S. (2021). Post-operative right ventricular failure after cardiac surgery: A cohort study. Front. Cardiovasc. Med..

[108] Rosa R.G., Roehrig C., Oliveira R.P.d., Maccari J.G., Antônio A.C.P., Castro P.d.S., Neto F.L.D., Balzano P.d.C., Teixeira C. (2015). Comparison of unplanned intensive care unit readmission scores: A prospective cohort study. PLoS ONE.

[109] Ponzoni C.R., Corrêa T.D., Filho R.R., Serpa Neto A., Assunção M.S., Pardini A., Schettino G.P. (2017). Readmission to the intensive care unit: Incidence, risk factors, resource use, and outcomes. A retrospective cohort study. Ann. Am. Thorac. Soc..

[110] Lee S.I., Koh Y., Huh J.W., Hong S.B., Lim C.M. (2022). Factors and outcomes of intensive care unit readmission in elderly patients. Gerontology.

[111] Zachrison K.S., Cash R.E., Adeoye O., Boggs K.M., Schwamm L.H., Mehrotra A., Camargo C.A. (2022). Estimated population access to acute stroke and telestroke centers in the US, 2019. JAMA Netw. Open.

[112] Plate J.D., Leenen L.P., Houwert M., Hietbrink F. (2017). Utilisation of intermediate care units: A systematic review. Crit. Care Res. Pract..

[113] Stelfox H.T., Hemmelgarn B.R., Bagshaw S.M., Gao S., Doig C.J., Nijssen-Jordan C., Manns B. (2012). Intensive care unit bed availability and outcomes for hospitalized patients with sudden clinical deterioration. Arch. Intern. Med..

[114] Engelman D.T., Ali W.B., Williams J.B., Perrault L.P., Reddy V.S., Arora R.C., Roselli E.E., Khoynezhad A., Gerdisch M., Levy J.H. (2019). Guidelines for perioperative care in cardiac surgery: Enhanced recovery after surgery society recommendations. JAMA Surg..

[115] Cowper P.A., DeLong E.R., Peterson E.D., Lipscomb J., Muhlbaier L.H., Jollis J.G., Pryor D.B., Mark D.B. (1997). Geographic variation in resource use for coronary artery bypass surgery. Med. Care.

[116] Eshghali M., Kannan D., Salmanzadeh-Meydani N., Esmaieeli Sikaroudi A.M. (2024). Machine learning based integrated scheduling and rescheduling for elective and emergency patients in the operating theatre. Ann. Oper. Res..

[117] Agasthi P., Ashraf H., Pujari S.H., Girardo M., Tseng A., Mookadam F., Venepally N., Buras M.R., Abraham B., Khetarpal B.K. (2023). Prediction of permanent pacemaker implantation after transcatheter aortic valve replacement: The role of machine learning. World J. Cardiol..

[118] Dell’Aquila M., Rossi C.S., Caldonazo T., Rahouma M., Harik L., Cancelli G., Ibrahim M., Van den Eynde J., Soletti G.J., Leith J. (2024). Machine learning versus logistic regression for permanent pacemaker implantation prediction after transcatheter aortic valve replacement—A systematic review and meta-analysis. J. Med. Artif. Intell..

[119] Ryan C.T., Zeng Z., Chatterjee S., Wall M.J., Moon M.R., Coselli J.S., Rosengart T.K., Li M., Ghanta R.K. (2023). Machine learning for dynamic and early prediction of acute kidney injury after cardiac surgery. J. Thorac. Cardiovasc. Surg..

[120] Malik M.I., Nedadur R., Chu M.W. (2025). An artificial intelligence and machine learning model for personalized prediction of long-term mitral valve repair durability. J. Thorac. Cardiovasc. Surg..

[121] Durand E., Sokoloff A., Urena-Alcazar M., Chevalier B., Chassaing S., Didier R., Tron C., Litzler P.Y., Bouleti C., Himbert D. (2019). Assessment of long-term structural deterioration of transcatheter aortic bioprosthetic valves using the new European definition: A multicenter French study. Circ. Cardiovasc. Interv..

[122] Koene R.J., Prizment A.E., Blaes A., Konety S.H. (2016). Shared risk factors in cardiovascular disease and cancer. Circulation.

[123] Légaré J.F., MacLean A., Buth K.J., Sullivan J.A. (2005). Assessing the risk of waiting for coronary artery bypass graft surgery among patients with stenosis of the left main coronary artery. Cmaj.

